# Germline-encoded neutralization of a *Staphylococcus aureus* virulence factor by the human antibody repertoire

**DOI:** 10.1038/ncomms13376

**Published:** 2016-11-18

**Authors:** Yik Andy Yeung, Davide Foletti, Xiaodi Deng, Yasmina Abdiche, Pavel Strop, Jacob Glanville, Steven Pitts, Kevin Lindquist, Purnima D. Sundar, Marina Sirota, Adela Hasa-Moreno, Amber Pham, Jody Melton Witt, Irene Ni, Jaume Pons, David Shelton, Arvind Rajpal, Javier Chaparro-Riggers

**Affiliations:** 1 Rinat R&D, Pfizer Inc., 230 East Grand Avenue, South San Francisco, California 94080, USA

## Abstract

*Staphylococcus aureus* is both an important pathogen and a human commensal. To explore this ambivalent relationship between host and microbe, we analysed the memory humoral response against IsdB, a protein involved in iron acquisition, in four healthy donors. Here we show that in all donors a heavily biased use of two immunoglobulin heavy chain germlines generated high affinity (pM) antibodies that neutralize the two IsdB NEAT domains, IGHV4-39 for NEAT1 and IGHV1-69 for NEAT2. In contrast to the typical antibody/antigen interactions, the binding is primarily driven by the germline-encoded hydrophobic CDRH-2 motifs of IGHV1-69 and IGHV4-39, with a binding mechanism nearly identical for each antibody derived from different donors. Our results suggest that IGHV1-69 and IGHV4-39, while part of the adaptive immune system, may have evolved under selection pressure to encode a binding motif innately capable of recognizing and neutralizing a structurally conserved protein domain involved in pathogen iron acquisition.

## Abstract (web summary)

*Staphylococcus aureus* can both cause disease in humans and be present with no discernible effect. Here, the authors look in detail at the memory immune response against a protein involved in iron acquisition.

## Graphical abstract

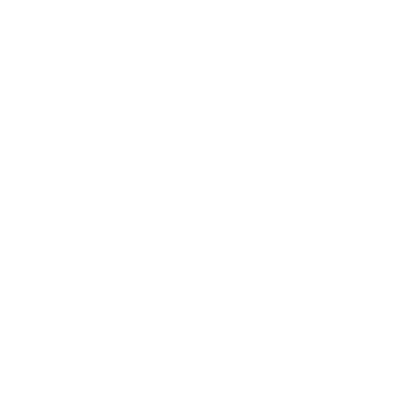

S*taphylococcus aureus* is a major human pathogen that can cause significant morbidity and mortality with a wide range of clinical manifestations^1^. These include bacteremia, pneumonia and infective endocarditis as well as osteoarticular, skin and soft tissue, and device-related infections^1^. The clinical burden is further exacerbated by the increasing occurrence of antibiotic resistance, in particular the raise of methicillin-resistant strains (MRSA)^2^. At the same time *S. aureus* is a human commensal that is carried persistently (20–30%) or transiently (≥50%) on the skin and in the nares of the general population^1^, with the majority of individuals never experiencing an overt infection episode. This remarkable commensal relationship, likely of evolutionary origins, affords the opportunity to study the immune response to a bacterial pathogen to which humans are exposed on a continuous or recurrent basis over their lifetime.

To explore the role of the immune response in this host/microbe interaction, we focused our attention on the mechanism used by *S. aureus* to obtain the iron necessary for colonization and pathogenesis^3^. *S. aureus* steals iron from haemoglobin, the most abundant iron source within vertebrates, through the concerted activity of the proteins in the iron-regulated surface determinant (Isd) locus^4^. IsdB in particular, a surface-exposed protein covalently anchored to the cell wall^5^, functions as a central component of this pathway by removing heme from haemoglobin and transferring it to other Isd proteins, which in turn import and degrade it to release iron in the bacterial cytoplasm^4^. IsdB contains two structurally conserved NEAT (NEAr iron Transporter) domains than bind haemoglobin and heme, respectively^6^,^7^,^8^,^9^. NEAT domains represent a structurally conserved heme-binding protein fold encoded in the genomes of several other Gram-positive human pathogens such as *Bacillus anthracis, Streptococcus pyogenes*, *Clostridium perfringens* and *Listeria monocytogenes*^10^,^11^. Importantly, *S. aureus* strains lacking IsdB or with IsdB mutants unable to bind haemoglobin, display a reduction in virulence in animal models of staphylococcal infection^6^,^12^. It was also previously shown that a recombinant anti-IsdB antibody was able to confer protection against *S. aureus* infections in animal models^13^.

High-serum titres against lsdB, as well as other *S. aureus* proteins, are readily observed in healthy adults^14^. While their biological significance and possible role in protection against infection remains to be elucidated, it has been shown that serum titres against IsdB are elevated during infection^15^,^16^. Therefore, to gain a better understanding of the functionality of these antibodies and to explore the relationship between the human immune system and the commensal pathogen *S. aureus*, we used single-B cell cloning, phage display libraries, high-throughput sequencing and epitope mapping^17^, structural and mutagenesis methodologies to characterize in detail the humoral immune response to the staphylococcal protein IsdB.

## Results

### Persistence of IsdB-reactive memory B cells

**Figure 1:**
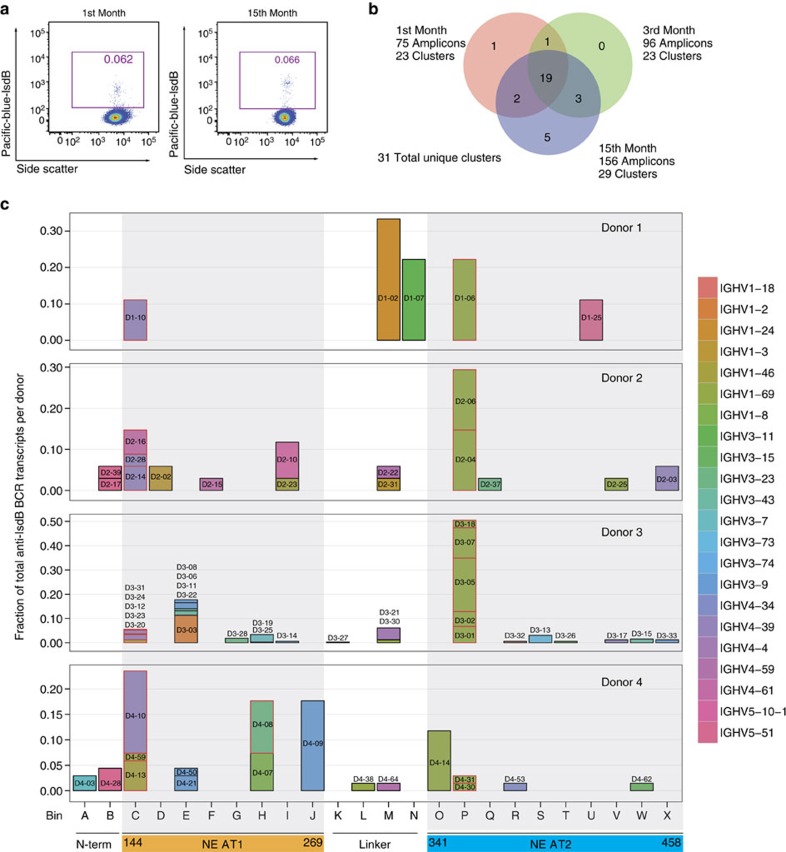
Characterization of anti-IsdB antibodies in the human memory B cell repertoire. (**a**) A persistent population of IsdB+ memory B cells from the peripheral blood mononuclear cells (PBMC) of a donor (D3) was observed over a 15-month period. By FACS, ∼0.06% of the IgM− CD19+ CD27+ memory B cells in the total memory B cell repertoire of this donor bind IsdB. (**b**) Most of the cloned BCR transcripts of the IsdB+ memory B cells collected at month 1, 3 and 15 are clonally related. BCR sequences from single-cell cloning of IsdB+ memory B cells were clustered based on heavy chain V-gene usage and CDR-H3 sequences. In total, we identified 31 unique clusters from donor D3 over the three collection time points. The Venn diagram shows that sibling clones within a cluster can be isolated at multiple time points. (**c**) Two distinct sets (bin C and bin P) of function-blocking antibodies specifically target NEAT1 and NEAT2, respectively. Single-cell cloning was performed at three different time points for donor D3 and one time each for donors D1, D2, and D4. In total, 75 unique antibodies targeting IsdB were identified and characterized. Shown here are the results of a comprehensive epitope binning analysis of 67 antibodies. Each reformatted clone is shown as a box and coloured according to its VH germline usage. The height of the box indicates the number of clustered BCR transcripts represented for each reformatted clone. There are in total 9, 34, 327, and 68 anti-IsdB single-cell BCR transcripts for D1, D2, D3 and D4, respectively. Each column of clones represents an epitope bin and this is overlaid on top of a linear representation of the IsdB molecule with NEAT1 in orange, and NEAT2 in blue. Clones that are able to fully block haemoglobin binding are outlined with a red box.

We first determined the presence of IsdB-reactive B cells in blood samples collected from a donor (D3) at months 1, 3 and 15 using flow cytometry (FACS), single-cell cloning (Supplementary Fig. 1) and high-throughput sequencing techniques. By FACS, we observed the persistence of a distinct IsdB-reactive memory B cell population (∼0.06%) within the IgM negative peripheral memory compartment (Fig. 1a). The majority of the IsdB-reactive memory B cells collected at three different time points expressed clonally related B cell receptor (BCR) transcripts: 25 of the 31 unique IsdB-reactive clusters obtained from this donor contained sibling transcripts isolated from at least two different time points (Fig. 1b). Longitudinal lineage analysis of the heavy chain variable region of these clusters indicates that the immune system maintains a repertoire of continually evolving antibodies against IsdB, presumably as a consequence of steady or intermittent exposure to low levels of antigen due to the commensal relationship between humans and *S. aureus* (Supplementary Fig. 2).

### Molecular characterization of the anti IsdB antibodies

**Figure 2:**
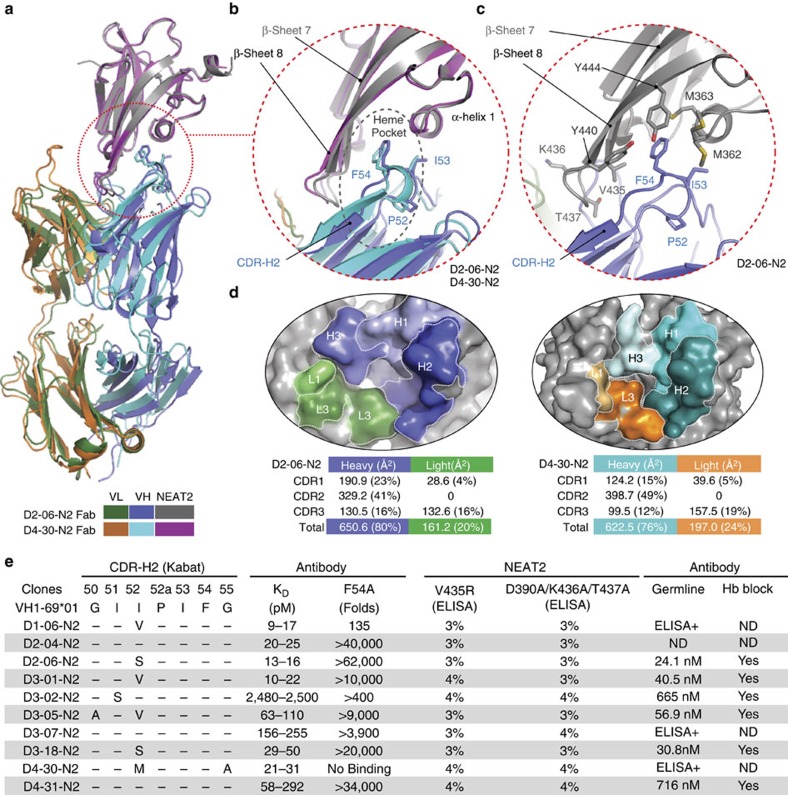
Germline-encoded binding of IGHV1-69 to the NEAT2 domain of IsdB. (**a**) Crystal structure of IGHV1-69-derived Fabs from two donors (D2-06-N2 and D4-30-N2) in complex with NEAT2. The Fabs of D2-06-N2 and D4-30-N2 show a near identical binding mechanism to NEAT2 as evidenced by the superimposed structures. To facilitate the crystallization process, a sandwiching Fab from an antibody (D3–13) that binds NEAT2 at a non-overlapping epitope was used. For clarity, the sandwiching Fab is removed from the figure, but is included in the Supplementary Data (Supplementary Fig. 10a,b). (**b**) Both IGHV1-69-derived antibodies use the conserved F54 on CDR-H2 to interact with the heme-binding pocket of NEAT2. The stem of the CDR-H2 loop also mediates major contacts with the β7-turn-β8 loop of NEAT2. (**c**) The heme pocket residues of NEAT2 which interact with the conserved F54 on CDR-H2 are highlighted in the complex with D2-06-N2. They are M362, M363 and F366 in α-helix 1, V435 on the β-strand 7, and Y440 and Y444 on the β-strand 8. (**d**) CDR-H2 dominates the interaction in terms of BSA in both structures. Structural analysis shows that 75–80% of the BSA is attributed to the heavy chain, and 20–25% to the light chain. In particular, the CDR-H2 contributes 41 and 49% of total BSA for the respective structures. (**e**) Mutational analysis confirms the structural data and demonstrates that all IGHV1-69-derived antibodies in this set bind NEAT2 with a similar mechanism. The K_D_ for all antibodies in this set and their respective F54A variants against IsdB NEAT2 were determined by SPR-based biosensor assays at 37 °C (K_D_ range, *n*≥2). Antibody binding to NEAT2 variants of V435R (heme-binding pocket) and D390A/K436A/T437A (β7-turn-β8 loop) was tested by ELISA (percentage binding relative to binding to wild type IsdB, one representative set of results out of three independent experiments is shown). Clones from each donor were reverted to VH germline sequence and tested for binding to NEAT2 by both ELISA and biosensor analysis, and ability to block haemoglobin binding. N.D. stands for not determined.

To investigate the nature of the interaction of these related antibodies with their target antigens we cloned the heavy and light chain BCR transcripts from single IsdB-reactive IgM− CD19+ CD27+ memory B cells obtained from the peripheral blood of four healthy donors with high-antibody titre to IsdB (D1-4) (Supplementary Figs 1 and 3). Overall, 75 unique antibodies representing 438 single-cell BCR transcripts were confirmed to bind both recombinant IsdB as well as IsdB on the surface of iron-starved *S. aureus* cells. Next, we determined their epitope binning, epitope mapping, affinity and ability to block haemoglobin binding to IsdB (Fig. 1c; Supplementary Data 1 and Supplementary Figs 4–7). We found the majority of the antibodies to be directed against epitopes on the conserved core of IsdB (NEAT1–Linker–NEAT2) (Fig. 1c; Supplementary Fig. 8a) with two prominent sets of antibodies (bins C and P) that block haemoglobin binding to IsdB, targeting NEAT1 and NEAT2, respectively. Antibodies belonging to both sets are found in all four donors (Fig. 1c; Supplementary Data 1). Surprisingly, among the antibodies that bind NEAT1, there is a strong bias (7 out of 12 antibodies) towards using the immunoglobulin heavy chain variable gene (IGHV) 4-39 and immunoglobulin kappa light chain variable gene (IGKV) (Supplementary Fig. 9a), whereas the antibodies that bind NEAT2 are invariably derived from the IGHV1-69 germline and IGKV light chains (Supplementary Fig. 9b), irrespective of the donor of origin. Remarkably, we found several of these antibodies to have affinities in the single digit pico-molar range at 37 °C (Fig. 2e; Supplementary Fig. 6).

### Characterization of IGHV1-69-derived NEAT2 binders (Bin P)

To elucidate the binding mechanism between the IGHV1-69-derived antibodies and NEAT2, we determined two crystal structures of Fabs from two different donors in complex with NEAT2 (Fig. 2a and Table 1; Supplementary Fig. 10a,b). Both structures, denoted by D2-06-N2 (3.22 Å) and D4-30-N2 (3.21 Å), surprisingly exhibited a nearly identical NEAT2 binding mode (Fig. 2a), with the heavy chain variable domain (VH), particularly complementary determining region (CDR)-H2, dominating the interaction in both structures (Fig. 2b). Specifically, the V-gene encoded CDR-H1 and CDR-H2 contribute 64% of the total buried surface area (BSA) for both structures (Fig. 2d). This heavy reliance on CDR-H1 and CDR-H2 is unusual for antibody/antigen interactions as highly diverse, VDJ recombination-generated CDR-H3s are typically considered to be the most important CDR for antigen binding^18^,^19^. The CDR-H2s of the Fabs engage the NEAT2 domain in two major modes. First, the β7-turn-β8 of NEAT2 slides into a groove at the interface of heavy and light chain variable regions, forming major contacts with the stem of the CDR-H2 loop. Second, F54 (Kabat numbering) of CDR-H2 protrudes into the hydrophobic heme pocket of NEAT2, made up of M362, M363 and F366 in the α-helix 1, V435 on the β7 and Y440 and Y444 on the β8 of IsdB (Fig. 2b,c). While this group of NEAT2 binding antibodies was initially found to block haemoglobin binding to IsdB, which likely occurs by steric hindrance as IsdB NEAT1 and NEAT2 are proposed to be spatially adjacent to each other based on their homology to the solved crystal structure of haemoglobin bound to IsdH NEAT2–linker–NEAT3 (ref. 20), the structural data reveals a second very effective way to block the activity of IsdB as antibody binding to the heme pocket precludes the possibility of concurrent heme binding^9^. Given the highly conserved nature of *S. aureus* NEAT2, particularly at the binding interface (Supplementary Fig. 8), we predict that these IGHV1-69-derived antibodies will be able to recognize and neutralize IsdB encoded by the vast majority if not all *S. aureus* strains (4,112 strains analysed in this study).

We next used mutagenesis to determine if the other eight IGHV1-69-derived antibodies in this set bind IsdB in a similar manner, as suggested by the fact that they all share the IGHV1-69 framework and a conserved F54 in CDR-H2. Figure 2e shows that a single mutation at the surface-exposed F54 position (F54A) resulted in >100-fold loss in affinity for all of the antibodies, highlighting that its importance for binding is shared by every antibody in this set. To further substantiate the common binding mode, we generated NEAT2 variants with mutations at the binding interface residues. Mutations in the heme pocket (V435R) and at the base of the β7-turn-β8 motif (D390A/K436A/T437A; Fig. 2c) consistently disrupted binding to every antibody in the NEAT2 binding set without affecting binding of antibodies that also bind NEAT2 but belong to different epitope bins (Fig. 2e). We observed slight differences in the extent to which mutations at several NEAT2 residues impacted the binding of the antibodies (Supplementary Fig. 11a,c), presumably due to subtle differences in how the distinct CDR-H3 and CDR-L3 of each antibody contribute to binding IsdB. Overall, the combination of structural and mutagenesis data indicates that IGHV1-69-derived antibodies from four donors bind NEAT2 in similar fashion by primarily using CDR-H2 germline residues.

Having established the prominent role of CDR-H2 in binding NEAT2 of IsdB, we determined if these antibodies have other commonalities by first examining the contribution of the individual CDR-H3 and J-region residues based on the two crystal structures and then by comparing the CDR-H3 amino acid usage of the 10 isolated binders to that of IGHV1-69-derived antibodies in memory repertoires of twelve healthy donors (Supplementary Fig. 12). For the heavy chain, we did not observe any common residue on CDR-H3 and JH that contributes substantially to the binding to NEAT2. Sequence analysis of the 10 binders also did not reveal any particular preference in immunoglobulin heavy chain joining segment (IGHJ) usage as IGJH1, 3 and 4 were all used (Supplementary Data 1). There seems to be a bias for charged residues (D, K or R) at position 95 and glycine at position 96, but both positions only have minor contributions to the overall binding based on the structures (Supplementary Fig. 12). As for the light chain, which overall contributes only 20–24% to the binding surface, there is no apparent preference for the IGKV or immunoglobulin kappa light chain joining segment (IGKJ) usage based on the 10 binders. Interestingly, the CDR-L3s of all the binders are 11 amino acids long, likely resulting from a direct fusion of IGKV and IGKJ genes. On the basis of the crystal structures, the two aromatic residues at position 94 and 96, which form a distinct motif (F/W-P-W/Y), are responsible for the majority of the CDR-L3 contribution to the binding. This motif was also found in another three binders, and a similar X-P-X motif was also found in four of the remaining five binders, suggesting a potential preference for light chain having a specific pattern at position 94–96 in pairing with IGHV1-69-derived heavy chain to bind NEAT2.

### Germline-reverted variants of IGHV1-69-derived antibodies

The shared binding mode of the IGHV1-69-derived antibodies led us to hypothesize that this germline has inherent potential to recognize the NEAT2 domain of IsdB. This hypothesis was tested by reverting the heavy chain V-gene region (framework 1 to framework 3) of multiple clones from each donor to their respective germline precursor sequences and testing their binding to IsdB NEAT2 by ELISA and biosensor. While we observed significant losses in monovalent affinity for all the germline-reverted clones (>600 fold compared with the originally isolated clones), all of them were still able to bind IsdB NEAT2 by ELISA with four germline-reverted clones, D2-06-N2, D3-01-N2, D3-05-N2 and D3-18-N2, having surprisingly high-monovalent affinity (24 nM–60 nM) to NEAT2 (Fig. 2e). In contrast, non-matured antibodies from naïve B cells typically bind antigen with high micro-molar affinity and binding can only be reliably detected in avidity-driven assays^21^,^22^. This data further supports the hypothesis that the IGHV1-69 germline possesses inherent capability to bind IsdB NEAT2. Also, these germline-reverted antibodies retain the ability to block haemoglobin binding (Fig. 2e; Supplementary Fig. 7).

### Allelic preference of IGHV1-69-derived antibodies

**Figure 3:**
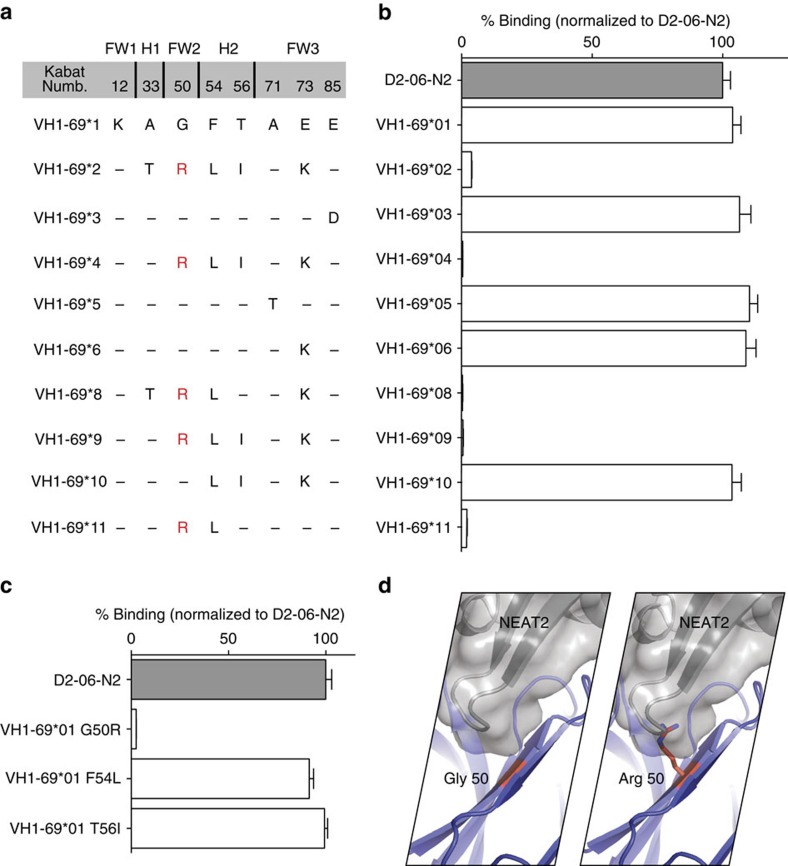
Allelic specificity of IGHV1-69-derived NEAT2 binders. (**a**) Amino acid differences among functional alleles of IGHV1-69. (**b**) The VH of clone D2-06-N2 was germline-reverted to all alleles with amino acid differences, and tested for binding to IsdB by ELISA. All alleles with a G50R substitution lost binding. ELISA data is an average of three independent experiments. Error bars are defined as s.d. (**c**.) Three individual variants (G50R, F54L and T56I) of D2-06-N2 (IGHV1-69*01 germline-reverted) were generated and their binding to IsdB was tested. Only variant G50R showed significant loss of binding. ELISA data is an average of three independent experiments. Error bars are defined as s.d. (**d**) Analysis of the structure illustrates how a change from G to R (most frequent rotamer) at position 50 is expected to cause a steric clash in the binding to NEAT2.

Moreover, we determined that the binding of IGHV1-69-derived antibodies to IsdB is strongly influenced by the allelic variation at position 50 (Fig. 3a). We found that the presence of R50, in contrast to G50 or A50 as in the antibodies described here, completely abolished the binding (Fig. 3b,c), presumably due to the steric clash between the extended side-chain of R50 and the IsdB β7-turn-β8 of NEAT2 (ref. 23; Fig. 3d). Data from the 1000 Genomes Project show that the R50 polymorphism, which abolishes NEAT2 binding, is present in 38% of the IGHV1-69 allele with a predicted homozygous rate of 14% in the general population^24^. This raises the possibility that there is a population-level difference in the ability to neutralize NEAT2-mediated heme-iron acquisition and therefore different susceptibility to *S. aureus* infection^25^,^26^.

### Characterization of IGHV4-39-derived NEAT1 binders (Bin C)

**Figure 4:**
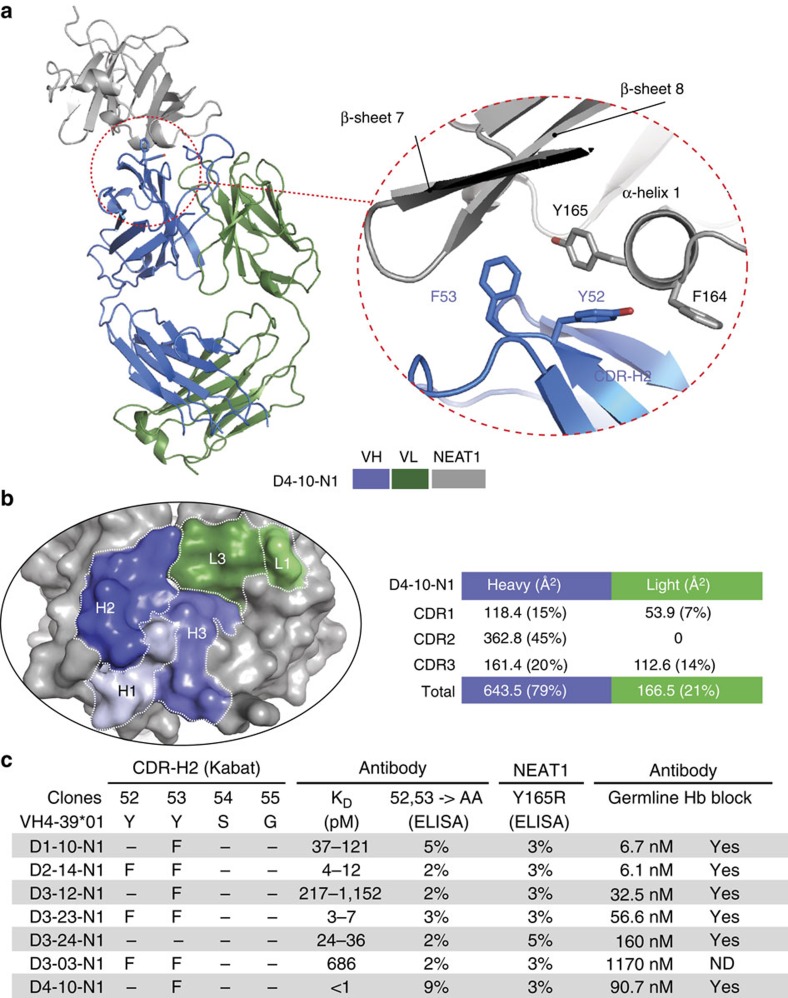
Germline-encoded binding of IGHV4-39 to the NEAT1 domain of IsdB. (**a**) Crystal structure of an IGHV4-39-derived Fab (D4-10-N1) in complex with NEAT1. The two aromatic resides (Y52 and F53) in CDR-H2 interact with the α-helix1 of NEAT1 which is normally involved in binding haemoglobin. IGHV4-39 CDR-H2 F53 of Fab D4-10-N1 protrudes into a hydrophobic pocket of NEAT1, which is structurally homologous to the heme binding pocket of NEAT2. Crystallization was facilitated by the use of a sandwiching Fab from an antibody (D3–19) that binds NEAT1 at a non-overlapping epitope (bin H). For clarity, the sandwiching Fab is removed from the figure but is included in the Supplementary Data (Supplementary Fig. 10c). (**b**) CDR-H2 dominates the interaction in terms of BSA. Structural analysis shows that 79% of the BSA is attributed to the heavy chain, and 21% to the light chain. The CDR-H2 contributes about 45% of total BSA. (**c**) Mutational analysis confirms the structural data and demonstrates that all IGHV derived antibodies in this set bind NEAT1 with a similar mechanism. The K_D_ for all antibodies in this set was determined by SPR-based biosensor binding analysis to recombinant full-length IsdB at 37 °C (K_D_ range, *n*≥2). The binding of antibody variants at positions 52 and 53 of CDR-H2 to wild type IsdB and the binding of antibodies to NEAT1 variant Y165R (α-helix 1) were evaluated by ELISA (percentage binding relative to binding between original isolated antibodies and wild type IsdB, one representative set of results out of three independent experiments is shown). Every clone was reverted to VH germline sequence and tested for binding to NEAT1 by biosensor analysis and ability to block haemoglobin binding. ND stands for not determined.

We also characterized a second class of antibodies that are derived from IGHV4-39 and bind to the NEAT1 domain of IsdB. We determined the crystal structure of the D4-10-N1 Fab in complex with NEAT1 (3.17 Å; Fig. 4a and Table 1; Supplementary Fig. 10c). The structure reveals that binding is again dominated by the heavy chain, particularly by CDR-H2, which contributes 45% of the BSA (Fig. 4b). Specifically, D4-10-N1 utilizes CDR-H2 residues Y52 and F53 to interact with residues Y165 of NEAT1, targeting the same binding region that is responsible for the interaction between haemoglobin and NEAT1 (ref. 6; Supplementary Fig. 13) and therefore providing a mechanistic explanation on how antibodies in this group block haemoglobin binding. Remarkably, CDR-H2 F53 protrudes into a hydrophobic pocket of NEAT1 that is structurally homologous to the heme pocket of NEAT2. Therefore this resembles the IGHV1-69 CDR-H2 interaction with NEAT2 (Supplementary Fig. 14). All antibodies in the NEAT1-binding group have a conserved aromatic residue (Y or F) at positions 52 and 53, and lost binding to NEAT1 when these residues were mutated to A (Fig. 4c). Correspondingly, mutations of NEAT1 at residue Y165 abolished binding for every antibody in this group without disrupting the binding of antibodies that also bind NEAT1 but belong to different epitope bins (Fig. 4c and Supplementary Fig. 11b,d). Collectively, the structural and mutational data strongly suggest that all of the antibodies in this set interact with NEAT1 in a similar fashion. Similar to NEAT2, the sequence of NEAT1 is also highly conserved (Supplementary Fig. 8), therefore we expect these IGHV4-39-derived antibodies to be able to recognize and neutralize IsdB encoded by the vast majority if not all *S. aureus* strains.

Among the seven IGHV4-39-derived NEAT1 binders there are no apparent preferences on the usage of IGKV germlines, IGKJ and IGHJ regions (Supplementary Data 1). Analysis of the amino acid usage in CDR-H3 (Supplementary Fig. 15) revealed a strong underrepresentation of R at position 94, as S, T and K were used instead. This may affect the typical salt-bridge connection between R94 and D101, which structurally supports the CDR-H3 loops. Four out of the seven binders are missing the typical pairing of K/R94 and D101. In addition, we also observed that P or G, which can alter the backbone conformation, are preferred at position 95. Therefore it is plausible that even though both residues do not mediate direct binding based on the crystal structure, they could co-operate to uniquely orient the CDR-H3 residues in these IGHV4-39-derived NEAT1 binders. Charged residues were also found preferentially at position 99 and 100 among the binders. However, both residues do not have any direct contact with NEAT1 in the crystal structure of D4-10-N1. The sequence/structure analysis did not reveal any common contact residue among the CDR-H3s of the seven IGHV4-39- IGKV-derived binders.

### Germline-reverted variants of IGHV4-39-derived antibodies

**Figure 5:**
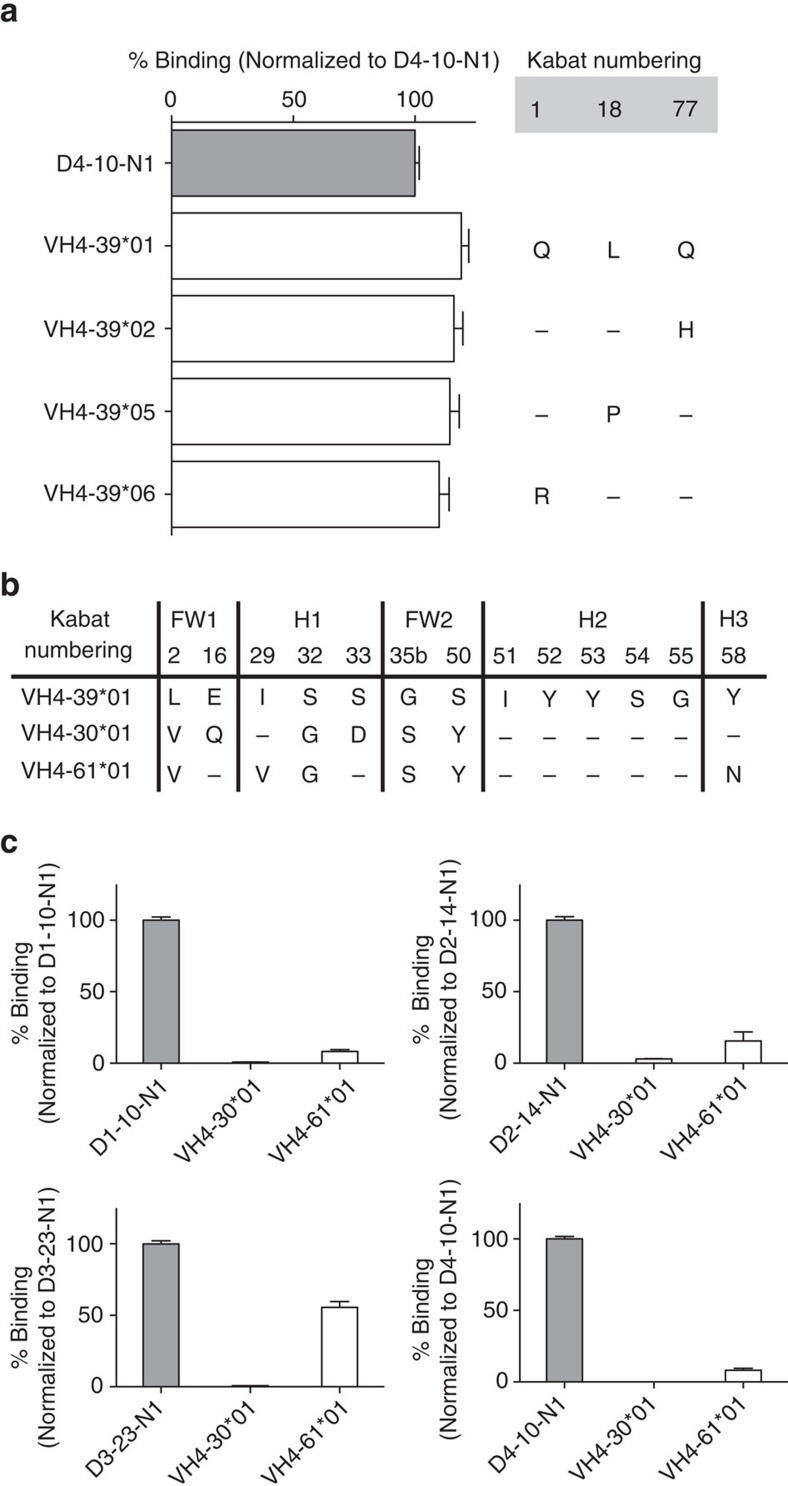
Germline and allelic specificity of IGHV4-39-derived NEAT1 binders. (**a**) Clone D4-10-N1 was reverted to all allelic variants with amino acid differences relative to IGHV4-39*01. No differences in IsdB binding were observed. Results shown are an average of three independent experiments. Error bars are defined as s.d. (**b**) IGHV4-39*01 has high sequence homology to IGHV4-30*04 and IGHV4-61*01 (they only differ by six amino acids in the variable region), and they all have the critical Y52 and Y53 residues in the CDR-H2. (**c**) The VH of four clones, one from each donor, were reverted to both IGHV4-30*04 and IGHV4-61*01, and their binding to IsdB was tested by ELISA. All IGHV4-30*01-derived variants were unable to bind IsdB, while most of the IGHV4-61*01-derived variants exhibited significantly loss of binding. ELISA data is an average of three independent experiments. Error bars are defined as s.d.

Given the prominent role of the IGHV4-39 germline-encoded CDR-H2 in binding IsdB NEAT1, we next measured the affinity of heavy chain V-gene germline-reverted (framework 1–framework 3) antibodies for all IGHV4-39 antibodies. Remarkably, all IGHV4-39 germline-reverted antibodies exhibited very high affinities (with K_D_ values at 37 °C in the single- to triple-digit nanomolar range; Fig. 4c), supporting the idea that the IGHV4-39 germline has intrinsic potential for recognizing IsdB NEAT1. These germline-reverted antibodies can also block haemoglobin binding (Fig. 4c; Supplementary Fig. 7). This feature appeared to be specific for IGHV4-39, as reverting selected antibodies to two other highly homologous germlines^23^, IGHV4-30*04 and IGHV4-61*01, resulted in significant loss of binding for the antibodies evaluated (Fig. 5). Unlike the IGHV1-69 NEAT2 binders, allelic variation did not appear to affect the ability of IGHV4-39-derived clones to bind NEAT1 (ref. 23; Fig. 5).

### IGHV4-39 encoded NEAT1 binders from naïve B cells

To expand the breadth of our findings we first tested serum samples from 36 donors (including the original 4 donors) against the two NEAT domains of IsdB and show that there are detectable titres against both NEAT domains (Supplementary Fig. 16). Moreover, these titres were reduced by pre-blocking the NEAT domains with antibodies that bind the haemoglobin and heme-binding sites, suggesting that antibodies that target the functional domains of IsdB are present in the serum of all donors tested (Supplementary Fig. 16).

**Figure 6:**
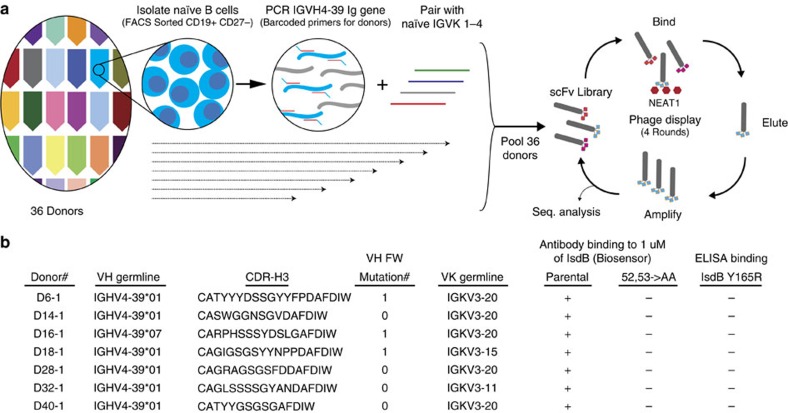
Naïve IGHV4-39-derived antibodies from naïve B cells. (**a**) Schematics of naïve IGHV4-39 antibody phage library generation. CD19+ CD27− IgM+ naïve B cells were isolated individually by FACS from 36 donors. Total Ig RNA was converted into cDNA using an IgM specific reverse primers, and then uniquely barcoded IGHV4-39 primers for each donor were used to selectively amplify the IGHV4-39 VH gene from the cDNA. The amplified VH genes were then pooled together and paired with the light chain variable genes from IGKV families 1–4 amplified from the same set of donors to generate the single-chain Fv library. Antibody libraries were then displayed on phage and 4 rounds of panning against recombinant IsdB NEAT1 were performed. (**b**) Binding characterization of IGHV4-39 encoded naïve NEAT1-binding antibodies from seven different donors. Heavy and light chain germlines usage, CDR-H3 sequence identities and number of variable heavy chain framework nucleotide mutation of the seven NEAT1 binders are shown. The observed single framework mutation in selected clones may have been introduced by the amplification process during library generation. The binding of the parental antibodies and their Y52A/Y53A variants to full-length IsdB was determined by SPR-based biosensor binding analysis at 37 °C. The binding of the parental antibodies to the full-length IsdB Y165R variant was determined by ELISA (*n*=2).

Next, given that the majority of the IsdB NEAT domain binding was primarily driven by germline-encoded CDR-H2, we investigate if antibodies from naïve B cells can recognize IsdB in a similar manner as the one described above and asked if such antibodies could be found in additional donors. Using individually barcoded IGHV4-39 specific primers, we selectively amplified the IGHV4-39 variable heavy chain gene from the cDNA of sorted CD19+ CD27− IgM+ naïve B cells of 36 individuals; this allowed us to unequivocally match binders with their respective donors. A single-chain Fv (scFv) phage display library was then constructed by pairing the individually barcoded IGHV4-39 VH gene with the pooled naïve IGKV families 1–4 genes from all donors (schematics are shown in Fig. 6a). Sequencing of the starting phage library confirmed that the heavy chain of more than 90% of the clones was encoded by IGHV4-39, with minor contaminations from other IGHV4 family members. After four rounds of panning against IsdB NEAT1 domains, the binding of 960 individual phage clones against IsdB and its variants was evaluated by ELISA. About 90% of the clones showed specific binding to full-length IsdB and IsdB NEAT1 domain. Sequence analysis determined that three of the phage clones with unique CDR-H3 (D14-1, D15-1 and D16-1) represented the majority of the binders (96%); this could be due to their superior affinity as scFv's (not determined) or to a growth bias introduced through the phage amplification process. Despite the presence of these three dominant clones, we were able to isolate a total of 16 clones with unique CDR-H3 sequences from 13 different donors (Supplementary Fig. 17).

The binding characteristics of the isolated clones were then evaluated. Remarkably all the unique phage clones lost binding to IsdB NEAT1 variant Y165R, suggesting that all the clones bind the haemoglobin-binding pocket on NEAT1 (Supplementary Fig. 17). Next, one clone from seven different donors was randomly selected and reformatted as IgG (Fig. 6b and Supplementary Fig. 18). Consistent with the phage binding results, all seven reformatted IGHV4-39-derived antibodies maintained the ability to recognize IsdB NEAT1 and all lost binding to the IsdB Y165R variant as measured by Elisa (Supplementary Fig. 18). We confirmed binding of these antibodies to IsdB in a monovalent based biosensor assay at 37 °C, while their Y52A/Y53A variants lost the ability to bind IsdB (Supplementary Fig. 18). Overall, these results showed that the naïve IGHV4-39 clones isolated from the naïve B cells of 13 additional donors bind IsdB NEAT1 in a manner similar and consistent with the binding interaction described above for the antibodies isolated from the memory B cells of four donors. This further strengthens the suggestion that IGHV4-39 possesses inherent affinity toward the NEAT1 domain of IsdB of *S. aureus*.

We next used the sequence information from these phage-derived NEAT1 binders to further examine the immunoglobulin gene use looking for any additional features that may be common to all IGHV4-39-derived binders. First, the naïve nature of these phage binders allows DH gene usage to be more reliably identified; we did not observe any preference for specific DH genes (Supplementary Fig. 17). Meanwhile, similar to what was observed for the NEAT1 binders isolated from memory B cells, we identified a strong underrepresentation of R at position 94 (G, S and T are used instead), as 14 of 16 phage binders (88%) do not have the typical salt-bridge pair of R94-D101. This is in contrast to the starting library, where 77% of the phage clones have R94 and D101 (Supplementary Fig. 19). In addition, the remaining two clones possessing the R94-D101 pair have P at position 95. This was also frequently observed in the memory B-cell-derived binders (Supplementary Fig. 15). Besides position 94 and 95, there is no apparent preference for amino acid usage in the CDR-H3 (Supplementary Fig. 19). Unexpectedly, IGHJ3 was exclusively used for all of the phage isolated binders. This strong bias of IGJH3 was not observed in our seven NEAT1 binders isolated from memory B cells, as all IGHJ1-6 genes were used. It is possible that this bias was introduced by displaying antibodies as scFv or by an inherent bias of the phage selection process. As for the light chain, IGKV3-20 was the most frequently used variable kappa light chain (VK) germline in these binders (11 out of 16), similar to what we observed from the memory B cells-derived NEAT1 binders (3 out of 7).

## Discussion

In this study, we characterized in detail the endogenous humoral response in healthy individuals against IsdB, a prominent molecule in the iron-acquisition pathway necessary for colonization and pathogenesis of the commensal bacterium *S. aureus*. We found that the human immune system maintains a sizable repertoire of continually evolving IsdB-reactive memory B cells by comparing the repertoire of a NEAT-2 binder over a 15-month period (Supplementary Fig. 2). For IsdB, high-serum titres are representative of antibodies that bind to a variety of epitopes on IsdB. Among them, we identified two prominent sets of neutralizing antibodies that target the specific fold of the highly conserved NEAT domains of IsdB with a dedicated, highly specific V-gene response for each NEAT domain, IGHV4-39 for NEAT1 and IGHV1-69 for NEAT2. Interestingly these neutralizing antibodies are not derived from the IGHV3 family which encode antibodies targeted by the *S. aureus* virulence factor Protein A^27^,^28^. These antibodies bind with very high affinity and neutralize the activity of IsdB by occupying the structurally homologous active regions on NEAT1 (Hb binding) and NEAT2 (heme binding) (Supplementary Fig. 14), respectively. The affinities of these antibodies, with dissociation constants measured at 37 °C in the low pM range in all four donors, are beyond the proposed *in vivo* affinity maturation limit of 100 pM to 1 nM (ref. 29). This perhaps results from the continuous or intermittent exposure to very low levels of antigen as a consequence of the commensal nature of *S. aureus*. Low antigen levels may only be recognized by B cell clones expressing these high affinity BCRs, thereby favoring their specific stimulation and subsequent selection in the context of the continually maturating B cell memory compartment.

We have isolated, by single-cell cloning, IGHV1-69-derived antibodies against NEAT2 and IGHV4-39-derived antibodies against NEAT1 from memory B cells from four donors and additional IGHV4-39-derived antibodies against NEAT1 from naïve B cells from 13 donors using a phage display approach. We show by a combination of structural and mutagenesis data that these antibodies bind the respective NEAT domains in a similar fashion. The binding interaction is primarily driven by the germline-encoded hydrophobic CDRH-2 motifs of IGHV1-69 and IGHV4-39. In fact, we also show that germline-reverted versions of these antibodies demonstrate specific binding to their respective NEAT domains and maintain the ability to block the interaction with haemoglobin. Sequence analysis of the CDR3s of the binders revealed positional preferences of certain amino acid residues, for example a strong underrepresentation of the canonical R at position 94 of the heavy chain for NEAT1 binders and the presence of aromatic residues at positions 94 and 96 of the light chain for NEAT2 binders. However, there is no conserved residue in CDR-H3 among the binders that mediates major contacts with the NEAT domains. Given the large size of the human antibody repertoire^30^,^31^ and the importance of the highly diverse CDR-H3 for binding^19^, antigen recognition that is predominantly driven by the invariant V-gene encoded CDR-H2 is rarely observed. Similarly, the occurrence of multiple antibodies isolated from different individuals that bind the same epitope with a similar mechanism is very rare and suggests a predetermined structural bias in the immune repertoire. Several examples have been reported in the literature to suggest a germline preference for antibody binding to certain antigens^32^,^33^. One noteworthy example is the recognition of the I/i antigen (*N*-acetyllactosamine) on red blood cells by IGHV4-34-derived IgM antibodies^34^,^35^. In absence of a co-crystal structure, mutagenesis studies suggested an unconventional binding mode as the germline-restricted interaction with the antigen is mediated by framework 1 residues and the C-terminal region of CDR-H3 (refs 36, 37). Another example of germline-restricted response is represented by antibodies against the capsular polysaccharide of *Haemophilus influenzae* type b. A strong preference for VH3 families usage was identified, in particular IGHV3-23 and IGHV3-15 (refs 38, 39). A subset of these antibodies has a binding interface characterized by IGHV3-23 with short CDR-H3 and IGKV2D-29 with an R residues inserted in the V–J junction^39^,^40^. A structural model and limited mutagenesis suggested potential interactions from all CDRs with the short CDR-H3 and long CDR-L1 forming the floor of a groove flanked by CDR1s and CDR2s of the heavy and light chain^41^. In both cases, in the absence of definitive structural characterization, the binding modes appear to be clearly different from the CDR-H2-driven interaction described in our study.

Detailed structural analysis of the binding mechanism is needed to illustrate the fine details of these germline-restricted recognitions; however, such structural data has largely not been available. The most prominent published body of work with detailed structural information is represented by two sets of IGHV1-69-derived broadly neutralizing antibodies that bind two distinct sites on the hemagglutinin molecule (HA) of the influenza virus. The first set of antibodies targets the receptor binding site on the globular head of HA^42^,^43^ (Supplementary Fig. 20) while the second set binds a structurally conserved epitope on the stem region of the molecule^44^,^45^,^46^ (Supplementary Fig. 21). Four antibodies from different donors that target the receptor binding site of HA, all with a heavy chain derived from IGHV1-69, were shown to recognize an overlapping epitope on the head of the molecule. This led to the suggestion of a IGHV1-69 germline preference for binding this region of HA. However, the structural data clearly shows that their binding modes are quite different from each other (Supplementary Fig. 20). This seems to stem from the fact that the binding interaction and orientation is driven by the different CDR-H3s of the different antibodies.

For the antibodies targeting the stem region of HA, IGHV1-69-derived antibodies isolated from three different donors were shown to consistently use a signature motif on CDR-H2 (I53/M53 and F54) and Y98 from CDR-H3 to target the same hydrophobic groove on HA^47^,^48^. Previous work has also shown that germline-reverted versions of these broadly neutralizing antibodies were able to engage HA^49^, suggesting that the IGHV1-69 CDR-H2 motif is well suited to recognize a specific epitope on the HA stem^50^. Our results parallel the work on antibodies against the stem region of HA and show that the germline-encoded structural motif on IGHV1-69 CDR-H2 is not only well suited to bind the hydrophobic heme pocket of IsdB NEAT2 of *S. aureus,* but is also the essential determinant of this binding (Figs 2d and 3b). Unlike the IGHV1-69-HA-stem binding mechanism, our data shows that CDR-H2 is so crucial for binding that IGHV1-69-derived antibodies from different healthy donors exhibit a nearly identical binding mechanism to NEAT2 (Supplementary Fig. 21). Moreover, to our knowledge, our work illustrates for the first time that the IGHV4-39 CDR-H2 motif is also particularly well suited to recognize the haemoglobin-binding domain of IsdB NEAT1, uncovering another example of a germline specialized in binding to a common antigen. Altogether, the fact that both sets of antibodies were found in all four donors tested and the results showing that the germline-reverted version of these antibodies maintain surprisingly high affinity for IsdB, further support the conclusion that the CDR-H2 motifs of IGHV1-69 and IGHV4-39 represent a particularly good fit to bind and neutralize the active sites on NEAT1 and NEAT2 of *S. aureus*, respectively. Because of the high degree of sequence identity among IsdB molecules encoded by *S. aureu*s strains, we expect these antibodies to be broadly neutralizing across these strains. In addition, NEAT domains are structurally conserved with many homologues encoded in the genomes of Gram-positive bacteria in the phylum Firmicutes^10^ (Supplementary Fig. 22), therefore we speculate that IGHV1-69 and IGHV4-39 antibodies against other significant human pathogens such as *Bacillus anthracis*, *Streptococcus pyogenes*, *Clostridium perfringens* and *Listeria monocytogenes* may be already present in humans, or may be induced on antigen exposure.

Overall, our study reveals that two human germlines, IGHV1-69 and IGHV4-39, have inherent affinity against the specific folds of structurally conserved NEAT domains of a bacterial commensal pathogen. The results expand the concept of germline-restricted usage of antibodies suggested mostly for viral pathogens^50^,^51^,^52^ and extend this to a bacterial pathogenic protein. In addition, our work also illustrates for the first time how germline V-gene encoded residues can be so dominant in driving antibody binding that resulting antibodies from different individuals exhibit nearly identical binding mechanism. The data suggest that existing human V-genes may represent not only V(D)J recombination scaffolds for the antibodies of the adaptive repertoire, but also innate-like proto-receptor scaffolds to recognize certain unique structural motifs presented by infectious pathogens, such as the influenza virus and *S. aureus*. This may allow a proportion of the adaptive immune repertoire to activate rapidly and provide protection against a pathogen at the earliest encounter, without the time required for lengthy affinity maturation. Given the presumptive evolutionary advantage of these responses^53^, it is possible that pathogens to which humans are exposed seasonally or recurrently due to a commensal relationship may have exerted evolutionary pressure to promote the retention or expansion of specific V-gene segments in the human repertoires.

## Methods

### Isolation and clustering of anti-IsdB antibodies

Blood samples of 50–100 ml were collected from healthy consented donors. Drawing of blood samples was approved by the Pfizer Institutional Review Board. The blood sample was first diluted 1:1 with PBS/2% FBS/1 mM EDTA and centrifuged at 120*g* for 10 min with the brake off. The plasma fraction was removed and replaced with an equivalent volume of PBS/2% FBS/1 mM EDTA. The sample was further diluted 1:1 with PBS/2% FBS/1 mM EDTA and layered on top of Ficoll-Paque PLUS (GE Healthcare). After centrifugation at 2,000 r.p.m. for 20 min with brake off, PBMC were collected from the interface and washed twice with PBS/2% FBS/1 mM EDTA. ACK lysis buffer (Thermo Fisher Scientific) was added to remove the red blood cells. After washing the cells with PBS/2% FBS/1 mM EDTA, B cells from the PBMC were enriched using EasySep Human Pan-B Cell Enrichment kit (Stemcell Technologies) according to the manufacturer's protocol. After the enrichment, B cells were washed with PBS/2% FBS/1 mM EDTA and labelled at 8 °C for 2–4 h with anti-human CD3-PerCP/Cy5.5 (1:20 dilution, UCHT1), anti-human CD16 PerCP/Cy5.5 (1:20 dilution, 3G8), anti-human CD19-AlexaFluor488 (1:20 dilution, HIB19), anti-human IgM-Phycoerythrin (1:20 dilution, MHM-88), anti-human CD27-Allophycocyanin (1:20 dilution, O323), 7-AAD (1:100 dilution) (all from Biolegend) and 40 nM recombinant IsdB conjugated to Pacific-blue according to the manufacturer's protocol (Thermo Fisher Scientific). Cells were then pelleted, washed and resuspended in PBS/2% FBS for FACS sorting. CD3−, CD16−, 7AAD−, IgM−, CD19+, CD27+, IsdB+ memory B cells were either bulk sorted for high-throughput sequencing or single-cell sorted into 96-well PCR plates for cloning. Individual cells were collected into each well of a 96-well plate containing 3.5 μl of Quick extraction buffer (Epicenter Bio) and immediately frozen over dry ice. Reverse transcription mixture containing 0.5 μl of reverse transcription primers mix (Supplementary Table 1), 5 μl of 2x reaction buffer and 1 μl of enzyme mix from SuperScript III One-Step RT-PCR system (Thermo Fisher Scientific) was added to the cell solution and reverse transcription was carried out at 55 °C for 30 min. Then, 20 μl of PCR mixture containing 0.15 μM each of the leader region primer mix, 0.25 μM each of constant reverse primer mix (Supplementary Table 1), 10 μl of the 2 × reaction buffer and 0.5 μl of enzyme mix from the SuperScript III One-Step RT-PCR system were added directly to the tube, which contained 10 μl of the reverse transcription reaction product. PCR reaction conditions were 94 °C for 2 min, 40 cycles of 94 °C for 15 s, 55 °C for 30 s and 68 °C for 1 min, a final extension step of 68 °C for 5 min. Separate nested PCR reactions were then performed to further amplify the transcripts of heavy chain and light chain (Vkappa+ Vlambda) using Taq polymerase system (Thermo Fisher Scientific) according to the manufacturer's protocol. Specifically, 0.2 μM each of variable region forward primer mix and 0.3 μM each of constant region reverse primer mix (Supplementary Table 1), and 2 μl of the first PCR reaction product as template were used in the nested PCR. Reaction conditions were 94 °C for 2 min, 40 cycles of 94 °C for 15 s, 56 °C for 30 s and 72 °C for 1 min, and a final extension step of 72 °C for 10 min. PCR amplicons were then gel-purified and sequenced. DNA sequences were analysed using an in-house developed software that identifies V-gene usage, J gene usage and CDRs identities. Antibodies (mAbs) sequences were then further triaged through a clustering algorithm based on VH and VL gene usages, CDR-H3 length and amino acid composition to identify unique clones and potential cluster of sibling clones for recombinant antibody expression.

### High-throughput sequencing of memory B cells

High-throughput sequencing was performed as previously described^54^. In brief, total RNA was isolated from bulk sorted CD3−, CD16−, 7AAD−, IgM−, CD19+, CD27+, IsdB+ memory B cells (see above for labelling conditions) using RNeasy micro kit according to the manufacturing protocol (Qiagen). RNA quality was assessed using an Agilent Bioanalyzer. Total RNA was reverse-transcribed into cDNA using the SMARTer RACE kit according to the manufacturing protocol (Clontech) and cDNA was used as template for five IgG-VH and two IgA-VH PCR reactions. The PCR reaction was carried out using a modified 5′ SMARTer RACE 10 × Universal Primer Mix (UPM) (Clontech) with the Lib-L-specific adaptor (Roche) sequence attached to the short oligo in the UPM (5′- CCTATCCCCTGTGTGCCTTGGCAGTCTCAGCTAATACGACTCACTATAGGGC-3′) and a IgG or IgA isotype specific 3′ primers (IgG, 5′-GGGAAGACSGATGGGCCCTTGG -TGG-3′; IgA, 5′-CAGGCAKGCGAYGACCACGTTCCCATC-3′) with the Lib-L-specific adaptor (5′-CCATCTCATCCCTGCGTGTCTCCGACTCAG-3′) and a six-nucleotide barcode sequence attached at the 3′ end. The reaction was carried out for 31 cycles following the manufacturer's recommendation. Barcoded IgG-VH (∼640 base pairs (bp)) and IgA-VH (∼630 bp) transcript libraries were purified using AMPure XP (Beckman Coulter), quantified using PicoGreen (Thermo Fisher Scientific) and pooled at equimolar amounts. The final multiplexed library pools were subjected to emulsion PCR and unidirectional sequencing using the GS FLX Titanium Lib-L chemistry (Roche).

### Lineage analysis of the memory B cells

Lineage analysis and the tree topology were performed with the nucleotide sequences generated from 454 high-throughput sequencing of the donor's memory B cell repertoire and from the corresponding single-cell cloning. The VDJ segments and VH CDR3 of each clone were first identified by using VDJFasta^31^. Sequences were clustered into each clonal lineage using the VDJFasta single linkage method described previously^54^. For tree topology representation of the memory B cell repertoire, somatic hypermutations and isotype in each sequence were identified by using VDJFasta. Sequences were aligned with MUSCLE algorithm^55^. Identical sequences at the nucleotide level were collapsed to a single sequence. To avoid erroneous connections due to DNA amplification error, any less frequent clonal lineage member, which has connectivity of 1 and is only one nucleotide different from a more frequent neighbour, was grouped into the higher frequency neighbour. A lineage tree topology was then generated using the nucleotide alignment, somatic hypermutation level and isotype identity. The diagram was generated using Cytoscape.

### Expression and purification of mAbs and protein reagents

Human IgGs and Fabs were expressed using the Expi293 system (Thermo Fisher Scientific). Human IgGs were purified with MabSelect (GE Healthcare). Fabs were histidine tagged and purified with Ni Sepharose Excel (GE Healthcare). Isd protein sequences used in this study are based on that of *S. aureus* strain USA300. The genes of Isd proteins were directly synthesized by GeneArt (Thermo Fisher Scientific) and cloned into either the pET-20 or the pET-47 expression vector (Novagen). Isd proteins and variants (truncations and point mutants) were expressed in BL21 DE3 cells (Thermo Fisher Scientific) under the control of lac operator with an N-term histidine tag and C-term Flag Tag. These constructs were purified with Ni Sepharose Excel (GE Healthcare). The NEAT1 and NEAT2 domains utilized in crystallization were expressed with an N-terminal histidine tag followed by a HRV 3C site using the pET-47b vector (Novagen). The NEAT domains were purified with Ni Sepharose Excel, and then subjected to HRV 3C cleavage overnight at 4 °C for tag removal. Digested protein was then reapplied to Ni Sepharose Excel and the flow through was collected, removing the tag and undigested protein. NEAT domains were then complexed with their respective Fab (excess NEAT) and underwent size exclusion chromatography using a Superdex 200 column in AKTA (GE Healthcare). The sandwich Fab was later added at a 1:1 ratio to this complex. Human haemoglobin (Hb) was purified from fresh red blood cells obtained from Bioreclamation LLC. Briefly, haemoglobin from cell lysates was purified by anion exchange chromatography using Q-sepharose XL (GE healthcare), followed by size exclusion chromatography with Superdex 200 Prep Grade in AKTA (GE healthcare)^56^.

### Epitope binning

Epitope binning for the anti-IsdB mAbs was carried out as previously described^17^. Briefly, anti-IsdB antibodies were individually amine-coupled as single spots onto a SensEYE G-COOH (Ssens bv) sensor chip to generate a 96-mAb array using a continuous flow microspotter (CFM) (Wasatch Microfluidics Inc). The printed sensor chip was then docked into an surface plasmon resonance (SPR) imager reader (MX96, IBIS Technologies bv) to perform interaction analysis of an analyte's binding towards the entire array of 96 antibodies simultaneously. Epitope binning experiments were performed using a classical sandwich assay format where each binding cycle comprised three steps; 35 nM rIsdB was injected for 3 min, 20 μg ml^−1^ antibody analyte was injected for a further 3 min, and then the surfaces were regenerated using a 30-s injection of 75 mM phosphoric acid. Ninety-six mAb analytes were injected over a 96-mAb array per unattended run. Binding data were analysed in SPRint software v. 6.15.2.1 and analysed in Wasatch Microfluidics' binning software for heat map generation, sorting and node plotting.

### Enzyme-linked immunosorbent assay (ELISA)

Recombinant Isd proteins and their variants (purified as described above) or commercially available alpha-toxin (Calbiochem) and SEB (Toxin Technologies) at 2 ug ml^−1^ in PBS were coated directly onto 96-well Maxisorp, Nunc-immunoplates (ELISA plate, Thermo Scientific) by incubation overnight at 4 °C. Alternatively, the molecules were captured through their FLAG tag with a previously coated anti-FLAG antibody (F1904, Sigma-Aldrich) for 2 h at RT. Following blocking with PBS/1% BSA/0.01% Tween-20 (ELISA buffer) for one hour at RT, the plates were washed six times with PBS/0.05% Tween-20 on an automated plate washer. Diluted human serum samples or mAbs serial dilutions in ELISA buffer where the added and incubated with shaking for one hour at RT. Plates were washed as above and an HRP-conjugated goat anti-human IgG (H+L) (Jackson Immunoresearch, 109-001-003) diluted 1:15,000 in ELISA buffer was added and incubation continued with shaking for one hour at RT. After a final wash as above, the plates were developed by addition of 3,3′,5,5′-Tetramethylbenzidine (TMB) peroxidase substrate (KPL) and the colorimetric reaction was stopped by addition of 5% phosphoric acid (Aqua Solutions). Absorption was read at 450 nm on a Spectra MAX 340 plate reader (Molecular Devices).

### Biosensor assay to determine the Hb-blocking effect of mAbs

The anti-IsdB mAbs were tested for their ability to block the rIsdB/Hb interaction as previously described^17^. Briefly, mAbs were captured at 15 μg ml^−1^ via anti-species sensors (10 min), 32 nM rIsdB was bound (10 min) followed by 1 μM Hb. Anti-species sensors were regenerated with 75 mM phosphoric acid. An isotype-matched negative control mAb (against an irrelevant target) was used to assess any non-specific cross-reaction of rIsdB or Hb.

### Cell-based assay to determine the Hb-blocking effect of mAbs

The binding of human Hb to endogenously-expressed IsdB on *S. aureus* cells was used to assess the blocking effect of anti-IsdB mAbs as previously described^57^ with the following modifications. The *S. aureus* ΔSpA strain was used. The antibodies, at a concentration of 600 nM, were pre-incubated with *S. aureus* cells for 10 min at room temperature before purified human Hb was added to give a final Hb concentration of 150 nM. Final detection of Hb was performed by standard Western blotting with a biotinylated primary antibody (sheep polyclonal anti-Hb, biotinylated, Abcam ab95152, used at 2 μg ml^−1^) followed by a streptavidin-conjugated secondary reagent (Streptavidin-IRDye 800 CW, 1 mg ml^−1^; Odyssey 926–32230, 1:4,000 dilution). A Licor Odyssey system was used to image the blot and to quantify the band intensities, data were expressed as per cent of maximum Hb binding in absence of antibodies.

### Affinity measurements of anti-IsdB mAbs

Active concentrations of the recombinant IsdB antigens were determined using a calibration-free concentration analysis on a Biacore T200 biosensor equipped with CM5 sensor chip (GE Healthcare), as described previously^58^. This assay relied on the use of a high affinity anti-IsdB antibody that could be regenerated easily; thus D1–06 was chosen as the reaction surface and was amine-coupled onto flow cell 2 at a high density (8,700 RU) to promote mass transport limited binding. Flow cell 1 was left blank (activated and blocked, without any protein coupled) to serve as a reference surface. Surfaces were generated with 75 mM phosphoric acid. These experiments returned apparent activities of 31–34% for both the full-length recombinant IsdB and IsdB NEAT 2 domain, and these ‘active' concentrations were used as input values for the kinetic experiments instead of the ‘nominal' protein concentrations (as determined by light absorbance at A280 nm with appropriate extinction).

Kinetic experiments were performed in a running buffer of PBS/0.01% Tween-20 using a one-shot kinetic method^59^ on a ProteOn XPR36 equipped with GLC sensor chips (BioRad). The surfaces for these experiments were prepared at 25 °C. Briefly, ligand channels were minimally activated using a 2–3 min injection of a freshly mixed aqueous solution of 1-ethyl-3-(3-dimethylaminopropyl)carbodiimide (EDC) and sulfo-*N*-hydroxysuccinimide (SNHS) at final concentrations of 1 mM EDC and 0.25 mM SNHS, antibodies were amine-coupled for 3 min at 15 μg ml^−1^ in 10 mM sodium acetate pH 4.5 buffer, and excess reactive esters were blocked with a 3 min injection of 1 M ethanolamine pH 8.5. Final amine-coupled levels ranged from 400–1,000 response unit (RU) per antibody, with<3% variation along the six spots within each ligand channel. The temperature was then adjusted to 37 °C to study the interaction of full-length recombinant IsdB or IsdB NEAT2 domain as analytes, which were injected for 3min along the analyte channels as a five-membered serial dilution along with a buffer sample to provide an in-line buffer blank for data processing purposes. The dissociation phase was monitored for up to 4 h. Alternatively, the interaction analysis was performed in a 36-ligand array format using a kinetic titration injection methodology, as described previously^60^. The same surfaces were also used to study analytes in a short and long injection methodology. The top analyte concentration used for the kinetic experiments, regardless of the injection method used, was adjusted as appropriate for the antibodies being studied, and varied from active concentrations of ∼1 μM (for the weak affinity binders) to 20 nM (for the high affinity binders). Analyte injections were performed in duplicate and all experiments were repeated on different chips to generate up to three independent measurements per interaction. Binding data were analysed using ProteOn Manager software; the sensorgrams from the reaction spots were interspot-referenced and double-referenced and fit globally to a simple Langmuir model with mass transport to deduce the equilibrium dissociation constant (K_D_=k_d_/k_a_) for each rIsdB/antibody binding interaction.

Binding of the germline-reverted antibodies were performed on a Biacore T200 SPR biosensor (GE Healthcare). Briefly, an anti-human Fc sensor chip was prepared by activating all flow cells of a Biacore CM4 sensor chip with a 1:1 (v/v) mixture of 400 mM EDC and 100 mM NHS for 7 min, at a flow rate of 10 μl min^−1^. An anti-human Fc reagent (Southern Biotech 2014-01) was diluted to 50 μg ml^−1^ in 10 mM sodium acetate pH 4.5 and injected on all flow cells for 7 min at 20 μl min^−1^. All flow cells were blocked with 100 mM ethylenediamine in 150 mM Borate buffer pH 8.5 for 7 min at 10 μl min^−1^. The running buffer for this immobilization procedure was 10 mM HEPES, 150 mM NaCl, 0.05% (v/v) Tween-20, pH 7.4. Kinetics experiments were performed at 37 °C using a running buffer of 10 mM sodium phosphate, 150 mM NaCl, 0.01% (v/v) Tween-20, pH 7.4. Anti-IsdB mAbs were captured on downstream flow cells (flow cells 2, 3 and 4) at concentrations that ranged from 8 to 20 μg ml^−1^ at a flow rate of 10 μl min^−1^ for 2 min. Flow cell 1 was used as a blank reference surface. Following capture mAbs, analyte (buffer, or IsdB) was injected at 30 μl min^−1^ on all flow cells for 2 min. Multiple IsdB analyte concentrations were tested. The IsdB analyte concentrations were 1.6, 8.0, 40, 200 and 1,000 nM. After the analyte injection, dissociation was monitored for 5 min. Following analyte binding and dissociation all flow cells were regenerated with three 1-minute injections of 75 mM Phosphoric Acid. The double-referenced sensorgrams were fit globally to a 1:1 Langmuir with mass transport binding model using the Biacore T200 evaluation software.

### Crystal structure determination and analysis

IsdB NEAT1 or NEAT2 in complex with their respective Fabs were subjected to Index (Hampton Research), JCSG+ (Qiagen) and PEG/Ion (Hampton Research) crystallization screens using the Mosquito robot. A number of initial hits were refined using vapour diffusion followed by microbatch using Al's oil (Hampton Research). D2-06-N2 complex crystallized with 20% PEG 3350 and 0.1 M sodium citrate at pH 5.4. The D2-06-N2 crystal was flash frozen with liquid nitrogen using mother liquor with 20% glycerol. D4-30-N2 complex was crystallized with 15% PEG 10 K and 0.1 M Tris pH 8.0, and flash frozen with mother liquor with 20% ethylene glycol. D4-10-N1 complex crystallized with 17% PEG 3350 and 0.2 M ammonium citrate tri basic pH 7.0, and flash frozen with mother liquor with 20% glycerol. Data collection was performed at Advance Light Source beamline 5.0.2. (Lawrence Berkeley National Labs). Images collected were indexed and scaled with HKL2000 (ref. 61), and Phaser^62^ was used for molecular replacement. The models were further refined with a combination of Coot 0.7 (ref. 63), CCP4i 6.5.0 (ref. 64), Phenix 1.9 (ref. 65), and autoBUSTER^66^.

### Generation of naïve IGHV4-39-derived phage antibody library

Blood samples of 25–50 ml were collected from healthy consented donors and PBMC were isolated as described earlier. A total of 1.0–1.9 million of CD19+ (1:20 dilution, HIB19), CD27− (1:20 dilution, O323) naïve B cells were FACS isolated from the PBMC of each donor. Total RNAs from each donor were individually obtained using the RNeasy mini kit according to the manufacturing protocol (Qiagen). First strand cDNA was synthesized using a human IgM heavy chain constant reverse primer (5′-GAAGGCAGCAGCACCTGTGAG-3′) and a human IgK light chain constant reverse primer (5′-TGGAGGGCGTTATCCACCTTCC-3′) in a reverse transcriptase reaction (SuperscriptIII, Thermo Fisher Scientific). Light chain cDNA from all donors were pooled and variable kappa light chain genes were amplified as previously described using the VK family 1–4 primers and JK reverse primers^67^. Heavy chain cDNA from each donor was first individually amplified for 20 cycles using an IGHV4-39 specific leader region primer (5′-TTCCTCCTGCTGGTGGCG-3′) and an IgM constant region reverse primer (5′-AAGTGATGGAGTCGG GAAGGAAG-3′). PCR conditions were according to the manufacturing protocol of *Pfu Ultra* (Agilent). A uniquely barcoded nesting primer for each donor was generated by adding nine unique nucleotides combinations, which are the different combinations of codons encoding for amino acids glycine-glycine-serine, in front of each IGHV4-39 specific forward primer (5′-CAGCTGCAGCTGCAGGAGTC-3′). The IGHV4-39 VH gene from each donor was then individually amplified for 20 cycles using 2 μl of first PCR product as template, and the barcoded IGHV4-39 forward primer and JH reverse primers^67^. Pooled VK genes and VH genes were then sequentially ligated into a single-chain Fv antibody phage display vector. SS320 cells (Lucigen) were transformed with the assembled scFv library vector in thirty parallel 50 μl electroporation reactions according to the manufacturing protocol. The size of the starting library was ∼6 × 10^9^. Antibody-displaying phages were recovered with M13KO7 helper phage (New England Biolab) according to previously published methods^68^. Briefly, overnight phage cultures were spun down at 12,000 g for 15 min. Supernatant was collected and incubated with 1:5 volumes of PEG 8000/2.5 M NaCl (Teknova) at room temperature for 20 min. The mixture was then spun down at 15,000 g for 10 min. Supernatant was removed and PBS was added to dissolve the phage pellet. Dissolved phage solution was spun down at 15,000 g for 10 min to remove any insoluble material. Phage supernatant was used immediately or frozen at −80 °C for storage.

### Selection of NEAT1 binders from antibody phage library

For panning of phage library, 2–4 μg ml^−1^ of recombinant IsdB NEAT1 protein in PBS was first coated overnight at 4 °C onto 24 wells of a Maxisorp plate. Plates were then blocked with either Superblock or StartingBlock (Thermo Fisher Scientific). After washing off the blocking solution with PBS/0.05%Tween, 10^13^ phage particles (100 μl per well) in PBS/1% BSA/0.05% Tween were then incubated with plate-bound NEAT1 protein overnight at 4 °C in the first round. The amount of phage input were subsequently reduced to 5 × 10^12^ particles in round 2, 10^12^ particles in round 3 and 5 × 10^11^ particles in round 4. Phage incubations for round 2 to round 4 were performed at room temperature for 2–4 h. After phage incubation, plates were washed with PBS/0.05%Tween for 5–20 times. Bound phages were recovered by incubating the well with 120 μl of 100 nM HCl for 20 min and immediately followed by neutralizing with 16 μl of 1 M TRIS pH 9.2. The eluted phages were then used to infect XL-1Blue (Agilent) cells growing at log phase (OD ∼0.3–0.6) for phage propagation and subsequent round of panning^68^. After four rounds of panning, infected *E. coli* were plated on LB carbenicillin plates. For screening, single *E. coli* colony were picked and individually inoculated in growth media (2YT/100 ug ml^−1^ carbenicillin/10^9^ M13KO7) overnight at 37 °C to produce phage. Phage cultures were then spun down and one sixth dilution of the phage supernatant in PBS/0.5% BSA/0.05% Tween was used in ELISA to test the binding of the phage clone to NEAT1. Phage ELISA conditions were similar to the ELISA conditions described earlier, but anti M13-IgG-HRP conjugate (GE Healthcare, 27942101) was used as the detection reagent. Clones that were reactive to IsdB NEAT1 and not binding negative control proteins were then sequenced. Selected clones with unique HC sequences from different donors were reformatted as human IgG1 for further testing.

### Sequence analysis of NEAT domains of *S. aureus* IsdB variants

Protein sequences from 4,152 *S. aureus* genomes annotated as IsdB were downloaded from the March 2015 release of the PATRIC pathogen database^69^. Additional filtering was performed to exclude partial sequences and incorrect protein or taxonomic classification: sequences with lengths of outside of the range 645±30 amino acids were excluded as well as sequences from the isolates *S. aureus* F87619 and *S. aureus* M21126. A multiple sequence alignment of the remaining 4,112 filtered protein sequences was generated using the MUSCLE algorithm^55^. The alignment was manually refined for the sequences of isolates from *S. aureus* subsp. aureus E1410, *S. aureus* RF122 and *S. aureus* O11 using Jalview^70^. The conservation score was computed as the frequency of the most commonly aligned residue at each position in the alignment. The multiple sequence alignment of the representative NEAT domain sequences was generated using the MUSCLE algorithm^55^ with the default alignment parameters.

### Data availability

The accession number for the structures of D2-06-N2, D4-30-N2, and D4-10-N1 in the Protein Data Bank are 5D1Q, 5D1X and 5D1Z, respectively.

The data that support the findings of this study are available within the article or from the corresponding authors on request.

## Additional information

**How to cite this article:** Yeung, Y. A. *et al*. Germline-encoded neutralization of a *Staphylococcus aureus* virulence factor by the human antibody repertoire. *Nat. Commun.* **7,** 13376 doi: 10.1038/ncomms13376 (2016).

**Publisher's note:** Springer Nature remains neutral with regard to jurisdictional claims in published maps and institutional affiliations.

## Supplementary Material

Supplementary Information Supplementary Figures 1-22, Supplementary Table 1 and Supplementary Reference.

Supplementary Data 1 Binding epitopes and sequence identification of human anti-IsdB antibodies.

## Figures and Tables

**Table 1:** Data collection and refinement statistics (molecular replacement).

	**D2-06-N2 5D1Q**	**D4-30-N2 5D1X**	**D4-10-N1 5D1Z**
*Data collection*			
Space group	P 42 21 2	P 41	P 2 21 21
Cell dimensions			
*a*, *b*, *c* (Å)	121.3, 121.3, 193.2	111.3, 111.3, 105.2	115.2, 147.1, 164.9
α, β, γ (°)	90, 90, 90	90, 90, 90	90, 90, 90
Resolution (Å)	47.33–3.22	49.18–3.21	24.96–3.17
*R*_sym_ or *R*_merge_	9.6 (53.6)	11.1 (46.3)	15.6 (56.8)
*I*/σ*I*	20.4 (3.4)	10.8 (1.97)	7.6 (1.95)
Completeness (%)	98.8	98.9	97.9
Redundancy	6.6 (6.6)	3.3 (3.1)	3.5 (3.4)
			
*Refinement*			
Resolution (Å)	3.22	3.21	3.17
No. reflections	22,680	19,661	45,212
*R*_work_/*R*_free_	21.7/28.1	27.1/30.9	23.7/28.6
*No. atoms*			
Protein	7,583	6,684	15,717
Ligand/ion	—	—	—
Water	—	—	—
*B-factors*			
Protein	93.787	87.022	49.474
Ligand/ion	—	—	—
Water	—	—	—
*R.m.s. deviations*			
Bond lengths (Å)	0.012	0.009	0.012
Bond angles (°)	1.697	1.465	1.539

One crystal used for each structure. *Values in parentheses are for highest-resolution shell.
