# Supplementary material for: Germline-encoded neutralization of a Staphylococcus aureus virulence factor by the human antibody repertoire
Source: Nat Commun. 2016 Nov 18;7:13376. doi: 10.1038/ncomms13376 (PMC5120205; doi:10.1038/ncomms13376)
Supplement: Supplementary Information — Supplementary Figures 1-22, Supplementary Table 1 and Supplementary Reference. [file ncomms13376-s1.pdf]

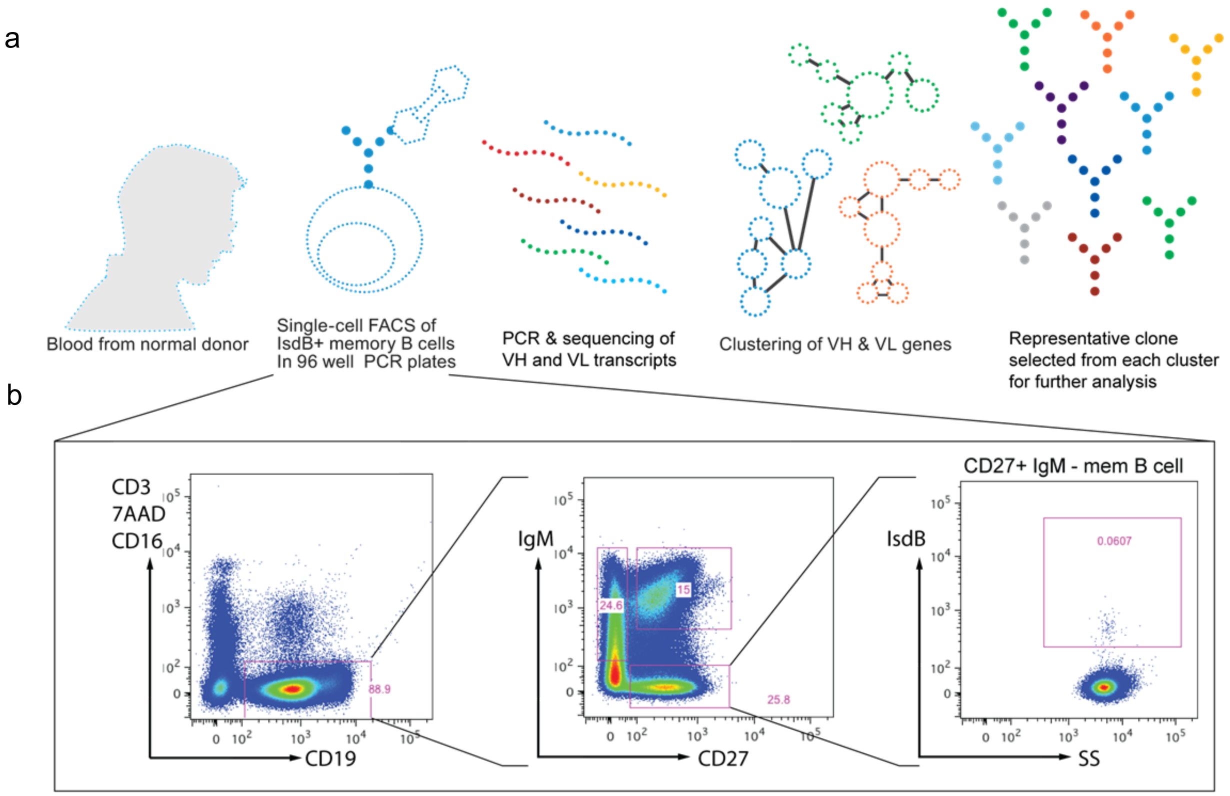

Supplementary Figure 1. Isolation of anti-IsdB antibodies from human B cells.

a) Schematic representation of how anti-IsdB antibodies were isolated. B cells were first enriched from the peripheral blood mononuclear cells (PBMC) of healthy donors and then sorted by their binding to Pacific-blue® conjugated recombinant IsdB. Individual IgM- CD19+ CD27+ IsdB+ memory B cells were FACS-sorted directly into a 96-well PCR plate. RT-PCR followed by two PCR amplification steps was applied to clone the heavy and light chain BCR variable domain transcripts (VH and VL) of each sorted cell. The amplicons were then sequenced and clustered based on heavy chain variable region gene usages and CDR-H3 sequences. A representative clone from each unique cluster was expressed recombinantly as human IgG for further characterization. b) Detailed gating strategy used to isolate anti-IsdB memory B cells. CD3- (T cell), CD16- (monocytes), 7AAD- (viability) and CD19+ (B cell) staining were first used to identify viable B cells and then IgM- CD27+ memory B cells within the total B cell population were selected. Finally, the population of memory B cells which binds Pacific-blue® conjugated IsdB was collected for cloning.

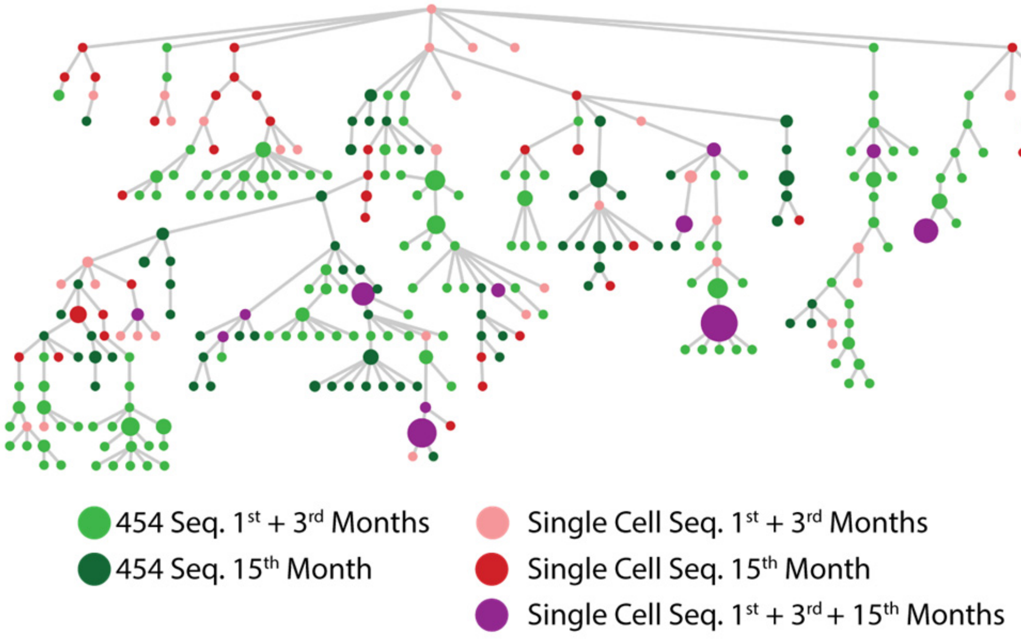

Supplementary Figure 2. Clonal lineage analysis of an IsdB-reactive memory B cell cluster using 454 high throughput sequencing and single cell cloning.

Lineage analysis of a single IsdB-reactive antibody cluster from donor D3 revealed continuous sequence permutation of the clone over a 15-month period. Each node stands for a unique VH nucleotide sequence within the clonal cluster and the node size represents the number of duplicate nucleotide sequences within each node (see methods for details on how clones were assigned into nodes). Nodes in the tree topology were organized to infer the path of somatic hypermutations and class-switching undertaken by the `clone based on the obtained sequences, with the least mutated at the top of the tree.

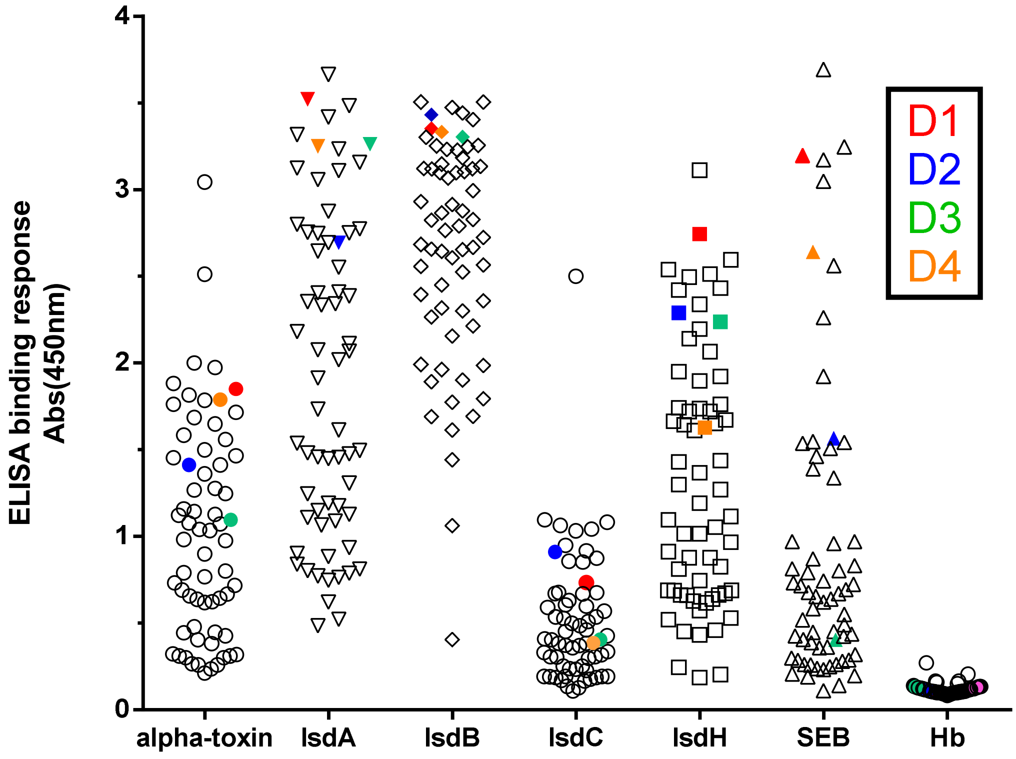

Supplementary Figure 3. Human serum titers against various *S. aureus* proteins.

The serum from 65 healthy donors with unspecified *S. aureus* carrier status was diluted 1:3000 and tested for the presence of antibodies against staphylococcal proteins by ELISA. IsdA, IsdB, IsdC and IsdH belong to the heme-iron acquisition pathway, alpha-toxin and SEB are secreted toxins and human hemoglobin (Hb) was used as negative control. The four donors chosen for single B cell BCR characterization (D1-4) are highlighted in different colors.

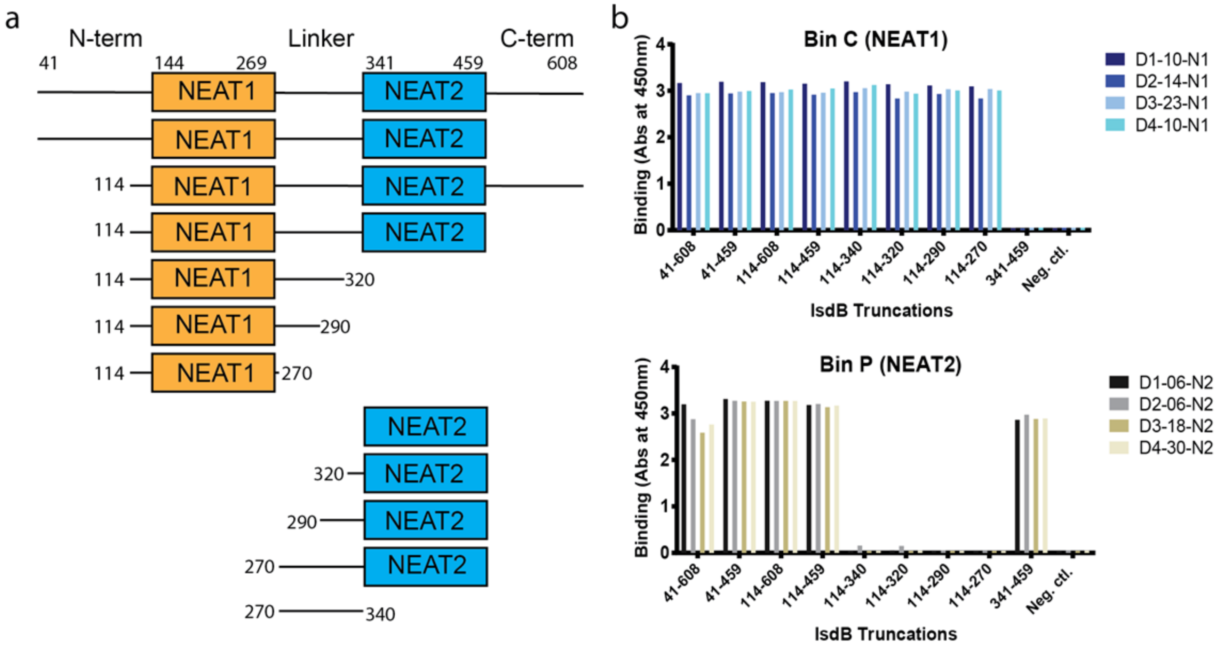

Supplementary Figure 4. Domain mapping of the anti-IsdB antibodies.

a) The IsdB ecto-domain structure can be divided into five main regions: N-terminus, NEAT1, Linker, NEAT2 and C-terminus. IsdB truncated versions were expressed and purified for antibody domain mapping. b) Representative clones from each epitope bin (Supplementary Table 1) were tested for binding to full-length IsdB and its truncated variants by ELISA. Recombinant *S. aureus* IsdA protein was used as the negative control (Neg. ctl.). Data for four representative clones in bin C (bind to NEAT1) and in bin P (bind to NEAT2) are shown.

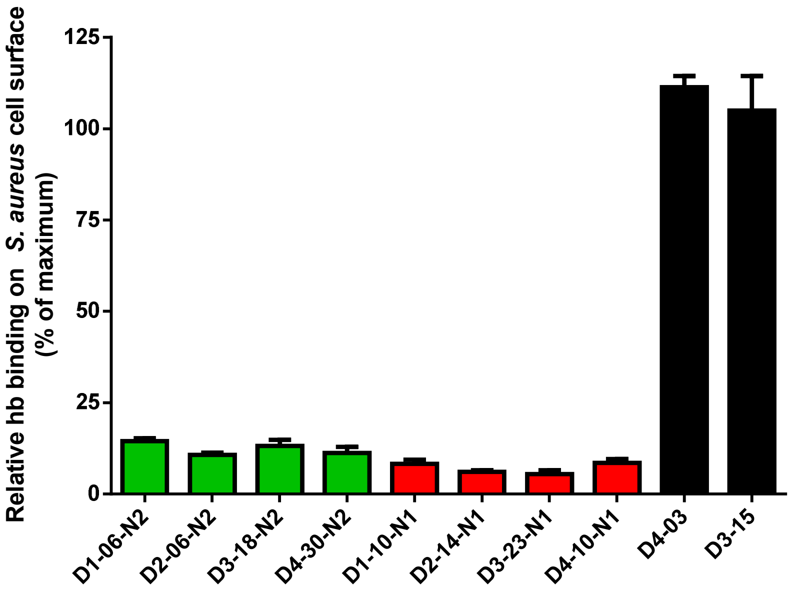

Supplementary Figure 5. Bin P antibodies that bind NEAT2 and bin C antibodies that bind NEAT1 effectively block hemoglobin binding to IsdB expressed on the surface of *S. aureus.*

Representative clones from each epitope bin (including all clones in bin C and P, i.e. the IGVH1-69 derived mAbs that bind NEAT2 and the IGVH4-39 derived mAbs that bind NEAT1) were tested for their ability to block hemoglobin binding to IsdB by biosensor and cell-based assays. For the biosensor assay, mAbs were captured on the sensor surface, recombinant IsdB was bound followed by the addition of hemoglobin (data not shown). For the cell-based assay, iron-starved *S. aureus* cells were sequentially incubated with mAbs and then hemoglobin. After rinsing the cells, bound hemoglobin was detected and quantified by western blot. Data shown is from one representative from each donor in bins C (IGVH4-39 mAbs) and P (IGVH1-69 mAbs) along with two controls (D4-03, bin A and D3-15, Bin W); average of three independent experiments.

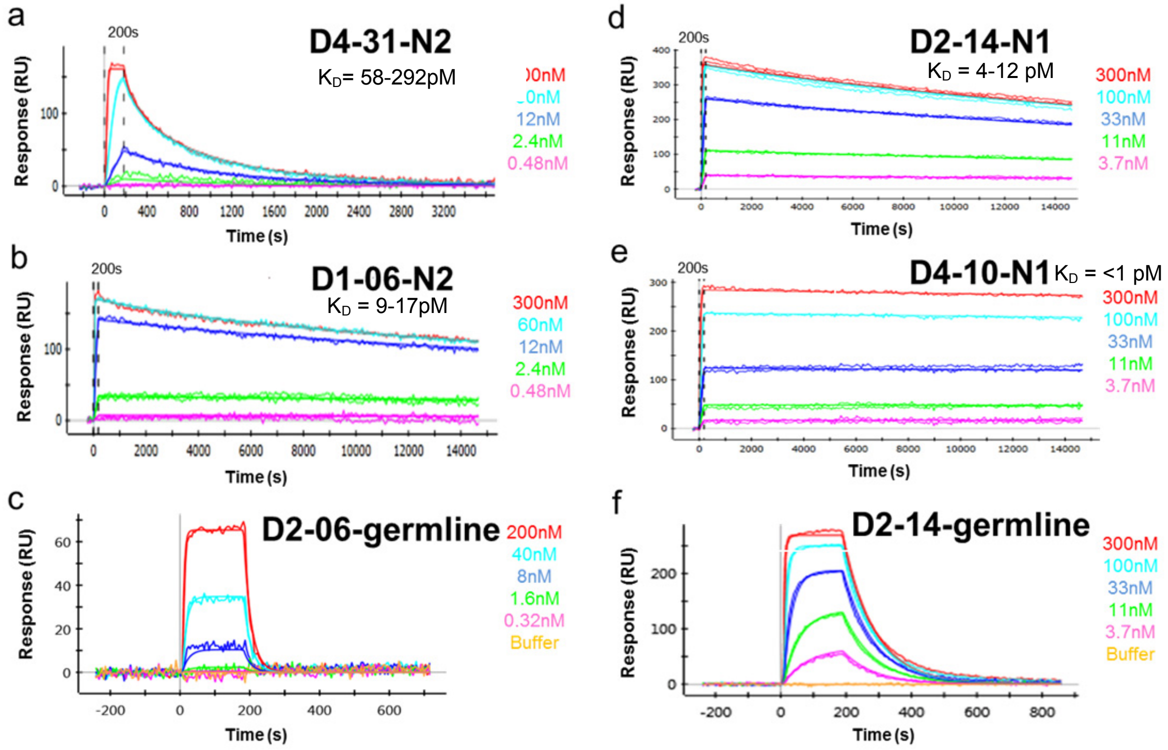

Supplementary Figure 6. Antibodies in bin C and P bind IsdB NEAT domains with high affinities.

a-c) Binding characterization of two antibodies in bin P (both IGHV1-69 derived) and a germline-reverted variant against recombinant IsdB NEAT2 domain. d-f) Binding characterization of two antibodies in bin C (IGHV4-39 derived) and a germline-reverted variant against recombinant IsdB. The binding interactions were analyzed using ProteOn XPR36. Briefly, antibodies and their variants were first conjugated on the surfaces of the sensor chip. Serial dilutions of the recombinant proteins at their "nominal" concentrations (shown here; see Methods section) were then injected over the antibody-conjugated surface for binding measurements. The experimental sensorgrams and the global fits are shown for each antibody.

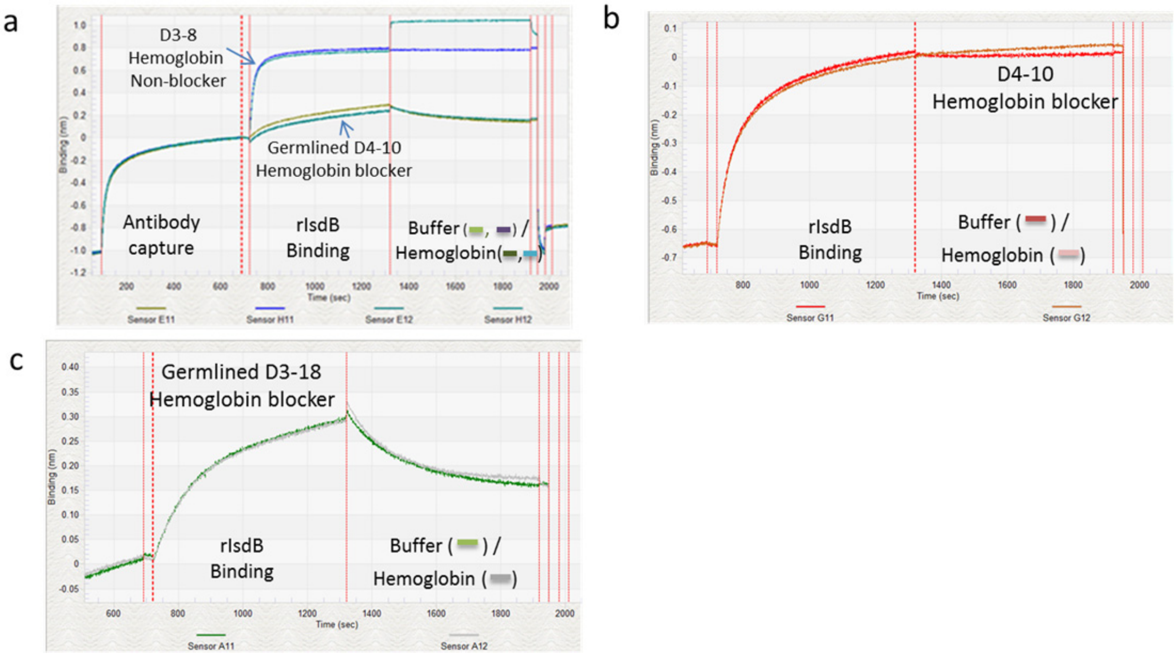

Supplementary Figure 7. Germline reverted antibodies block hemoglobin binding to IsdB.

Recombinant full length IsdB was first allowed to bind germline reverted or control antibodies captured on the Octet sensor chip. The antibody-IsdB complex was then incubated with either hemoglobin or buffer. Lack of hemoglobin binding to the antibody-IsdB complex gives a binding curve similar to the buffer-alone binding curve. Examples of a non-hemoglobin-blocking parental antibody D3-8 (a), a hemoglobin-blocking parental antibody D4-10 (b), and two hemoglobin-blocking germline reverted antibodies, germlined D3-18 (NEAT2) (c) and germlined D4-10 (NEAT1) (a) are shown.

a

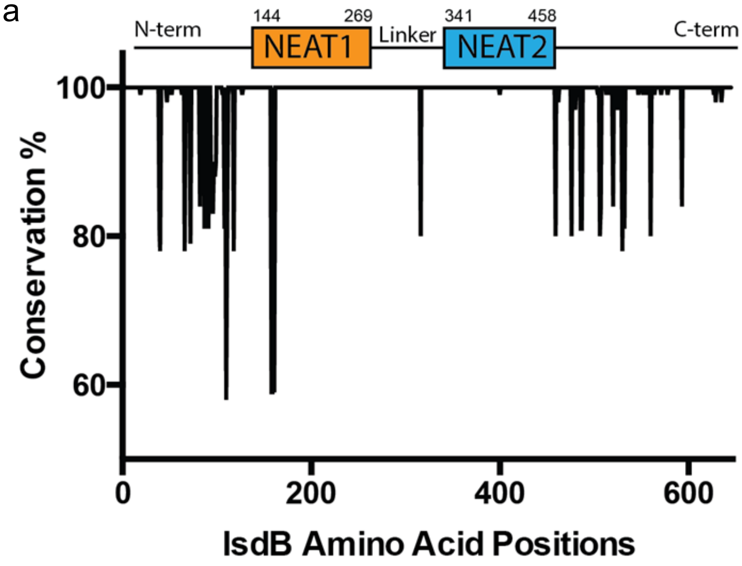

b

NEAT1 Position	NEAT1 Conseravtion	NEAT2 Position	NEAT2 Conseravtion
LYS 157	100%	MET 362	100%
ASP 159	41%	MET 363	100%
GLY 160	100%	THR 365	100%
THR 161	41%	PHE 366	100%
GLN 162	100%	ASN 388	100%
GLN 163	100%	ASP 390	100%
PHE 164	100%	TYR 391	100%
TYR 165	100%	LYS 407	100%
TYR 167	100%	ALA 409	100%
ALA 168	100%	LYS 410	100%
SER 169	100%	ASN 411	100%
GLN 190	100%	ASN 412	100%
SER 191	100%	VAL 433	100%
PHE 194	100%	VAL 435	100%
ASP 219	100%	LYS 436	100%
THR 240	100%	THR 437	100%
HIS 241	100%	ILE 438	100%
PHE 242	100%	ASP 439	100%
ASN 243	100%	TYR 440	100%
LYS 245	100%	TYR 444	100%
GLU 247	100%		
TYR 249	100%		

Supplementary Figure 8. The NEAT domain amino acid residues in contact with the antibodies are highly conserved among 4,112 *S. aureus* IsdB sequences.

a) Amino acid conservation across IsdB sequences obtained from 4,112 *S. aureus* strains (PATRIC database)The conservation percentage was computed as the frequency of the most commonly aligned residue at each position in the alignment. The core region of IsdB (NEAT1-linker-NEAT2) is highly conserved. b) Conservation percentage of the NEAT domain residues in contact with antibodies as determined by buried surface area (ePISA). Results show that the majority of the NEAT contact residues are highly conserved among the 4,112 IsdB sequences analyzed.

a

																													CDR-H1				
Kabat Numb.	1	2	3	4	5	6	7	8	9	10	11	12	13	14	15	16	17	18	19	20	21	22	23	24	25	26	27	28	29	30	31	32	33
VH1-69*1	Q	V	Q	L	V	Q	S	G	A	E	V	K	K	P	G	S	S	V	K	V	S	C	K	A	S	G	G	T	F	S	S	Y	A
D1-06-N2	-	-	-	-	-	-	-	-	-	-	-	-	-	-	-	-	-	-	T	-	-	-	Q	-	-	-	-	I	-	N	-	L	D
D2-04-N2	-	-	-	-	-	-	-	-	-	-	-	-	-	-	-	-	-	-	-	-	-	-	-	V	-	-	-	P	-	N	R	H	T
D2-06-N2	-	-	-	-	-	-	-	-	-	-	-	-	-	-	A	-	-	-	-	-	-	-	Q	-	-	-	-	S	-	-	T	R	T
D3-01-N2	-	-	-	-	-	-	-	-	-	-	-	-	-	-	-	-	-	-	-	-	-	-	-	S	-	-	-	N	-	N	T	H	G
D3-02-N2	-	-	-	-	-	-	-	-	-	-	-	-	-	-	-	-	-	-	-	-	-	-	-	-	-	-	-	-	-	-	N	-	-
D3-05-N2	-	-	-	-	-	-	-	-	-	-	-	-	-	-	-	-	-	-	-	-	-	-	-	-	-	-	-	-	-	N	T	N	G
D3-07-N2	-	-	-	-	-	-	-	-	N	-	-	-	-	-	-	-	-	-	-	-	-	-	-	V	-	-	-	-	-	-	T	-	S
D3-18-N2	-	-	-	-	-	-	-	-	-	-	-	R	-	-	-	-	-	-	-	-	-	-	-	-	-	-	-	S	-	T	R	F	V
D4-30-N2	-	-	-	-	-	-	-	-	S	-	-	-	-	-	-	-	-	-	T	L	-	-	-	-	-	-	D	-	-	N	T	H	T
D4-31-N2	-	-	-	-	-	-	-	-	-	-	-	-	Q	-	-	-	-	L	-	-	-	-	-	-	-	-	D	S	-	T	K	-	P

																					CDR-H2							
Kabat Numb.	34	35	36	37	38	39	40	41	42	43	44	45	46	47	48	49	50	51	52	52a	53	54	55	56	57	58	59	60
VH1-69*1	I	S	W	V	R	Q	A	P	G	Q	G	L	E	W	M	G	G	I	I	P	I	F	G	T	A	N	Y	A
D1-06-N2	-	-	-	L	-	-	-	-	-	H	-	P	-	-	-	-	-	-	V	-	-	-	-	P	-	K	N	-
D2-04-N2	-	-	-	-	-	-	G	-	-	-	-	-	-	-	V	-	-	-	-	-	-	-	-	A	-	K	N	-
D2-06-N2	-	-	-	L	-	-	-	-	-	-	-	-	-	-	-	-	-	-	S	-	-	-	-	A	-	-	-	-
D3-01-N2	V	T	-	-	-	-	-	-	-	-	-	-	-	-	-	-	-	-	V	-	-	-	-	A	T	K	S	-
D3-02-N2	-	H	-	-	-	-	-	-	-	E	-	-	-	-	-	-	-	S	-	-	-	-	-	A	-	-	-	-
D3-05-N2	-	T	-	-	-	-	-	-	-	-	-	-	-	-	-	-	A	-	V	-	-	-	-	A	T	-	-	-
D3-07-N2	-	-	-	-	-	-	-	-	-	-	-	-	-	-	-	-	-	-	-	-	-	-	-	A	-	K	N	T
D3-18-N2	-	-	F	L	-	-	-	-	-	-	-	F	-	-	-	-	-	-	S	-	-	-	-	A	-	-	-	-
D4-30-N2	Y	-	-	-	-	-	-	-	-	-	R	-	-	-	I	-	-	-	M	-	-	-	A	A	S	K	S	-
D4-31-N2	-	-	-	-	-	-	-	-	-	-	-	-	-	-	-	-	-	-	-	-	-	-	-	A	T	-	-	-

Kabat Numb.	61	62	63	64	65	66	67	68	69	70	71	72	73	74	75	76	77	78	79	80	81	82	82a	82b	82c	83	84	85
VH1-69*1	Q	K	F	Q	G	R	V	T	I	T	A	D	E	S	T	S	T	A	Y	M	E	L	S	S	L	R	S	E
D1-06-N2	-	-	-	-	-	-	L	-	-	-	-	-	K	-	-	T	-	T	-	-	-	-	-	-	-	-	-	-
D2-04-N2	-	-	-	-	-	-	-	-	-	-	-	-	-	-	-	-	-	-	F	-	-	-	-	G	-	-	-	-
D2-06-N2	-	R	-	-	-	-	-	-	-	-	-	-	K	-	-	-	-	-	-	-	-	-	T	-	-	T	-	-
D3-01-N2	-	-	-	-	-	-	-	S	L	-	-	-	-	-	-	T	-	-	-	-	-	-	R	G	-	T	Y	-
D3-02-N2	-	-	-	-	D	-	-	-	-	-	-	-	-	-	-	-	-	-	-	-	-	-	-	-	-	T	-	-
D3-05-N2	-	R	-	-	-	-	-	-	-	-	-	-	-	-	-	T	-	-	-	L	-	F	-	-	-	T	P	-
D3-07-N2	-	-	-	-	D	-	-	-	-	S	-	-	-	-	-	-	-	V	-	-	-	V	K	-	-	I	-	-
D3-18-N2	-	-	-	-	-	-	-	-	-	-	-	-	-	-	A	-	-	-	-	-	-	-	-	-	-	T	-	D
D4-30-N2	P	H	L	-	D	-	L	-	-	-	-	-	-	A	-	R	-	-	-	-	-	-	T	-	-	T	-	D
D4-31-N2	-	-	-	-	-	-	-	-	-	-	-	-	-	-	S	T	-	-	-	-	-	-	-	-	-	-	-	-

												CDR-H3																				
Kabat Numb.	86	87	88	89	90	91	92	93	94	95	96	97	98	99	100	100a	100b	100c	100d	101	102	103	104	105	106	107	108	109	110	111	112	113
VH1-69*1	D	T	A	V	Y	Y	C	A	R																							
D1-06-N2	-	-	-	F	-	-	-	-	T	K	R	G	N	T	Y	G	.	.	F	D	S	W	G	Q	G	T	L	V	T	V	S	S
D2-04-N2	-	-	-	-	-	F	-	-	-	D	G	P	G	D	D	A	.	.	F	Q	I	W	G	Q	G	T	L	V	T	V	S	S
D2-06-N2	-	-	-	-	-	-	-	-	-	D	R	P	P	G	A	Q	.	.	F	D	T	W	G	Q	G	T	L	V	T	V	S	S
D3-01-N2	-	-	-	-	-	-	-	-	S	R	G	L	E	Y	K	N	W	G	L	E	F	W	G	Q	G	T	L	V	T	V	S	S
D3-02-N2	-	-	-	-	-	-	-	-	-	R	G	D	G	Y	N	N	W	G	F	D	F	W	G	Q	G	T	L	V	T	V	S	S
D3-05-N2	-	-	-	-	-	-	-	-	T	R	G	R	E	H	N	N	W	G	L	E	Y	W	G	Q	G	T	L	V	T	V	S	S
D3-07-N2	-	-	-	-	-	-	-	-	-	D	A	T	V	G	A			.	L	G	H	W	G	Q	G	T	L	V	T	V	S	S
D3-18-N2	-	-	-	-	-	F	-	-	-	D	S	Q	R	G	A	S	Y	G	F	Q	F	W	G	Q	G	T	L	V	T	V	S	S
D4-30-N2	-	S	G	-	-	-	-	-	-	D	G	R	G	A			.	.	L	Q	Y	W	G	Q	G	T	L	V	T	V	S	S
D4-31-N2	-	S	-	-	-	-	-	-	-	D	G	V	D	T	.	.	.	.	F	D	I	W	G	Q	G	T	M	V	T	V	S	S

b

																														H1					
Kabat Numb.	1	2	3	4	5	6	7	8	9	10	11	12	13	14	15	16	17	18	19	20	21	22	23	24	25	26	27	28	29	30	31	32	33	34	35
VH4-39	Q	L	Q	L	Q	E	S	G	P	G	L	V	K	P	S	E	T	L	S	L	T	C	T	V	S	G	G	S	I	S	S	S	S	Y	Y
D1-10-N1	-	V	-	-	-	-	-	-	-	-	-	-	-	-	-	-	-	-	-	-	-	-	-	-	-	-	-	-	V	T	T	G	-	-	F
D2-14-N1	-	V	-	-	-	-	-	-	-	-	-	-	-	-	-	-	-	-	-	-	-	-	S	-	-	-	-	-	V	R	-	-	-	-	-
D3-12-N1	-	V	-	-	-	-	-	-	-	-	-	-	-	-	-	-	-	-	-	-	-	-	-	-	-	-	-	-	-	R	-	-	-	F	-
D3-23-N1	-	V	-	-	-	Q	-	-	-	-	-	L	-	-	-	-	-	-	-	-	-	-	S	-	-	-	-	-	F	R	-	-	-	-	-
D3-24-N1	-	V	-	-	-	-	-	-	-	-	-	-	-	-	-	-	-	-	-	-	-	-	I	-	-	-	-	-	-	-	-	-	-	-	-
D3-31-N1	-	V	-	-	-	-	-	-	-	-	-	-	-	-	-	-	-	-	-	-	I	-	-	-	-	-	-	-	-	-	-	-	-	-	-
D4-10-N1	-	V	-	-	-	-	-	-	-	-	-	-	-	-	-	-	-	-	-	-	-	-	N	-	-	-	-	-	-	G	-	-	A	-	-

																					H2						
Kabat Numb.	35a	35b	36	37	38	39	40	41	42	43	44	45	46	47	48	49	50	51	52	53	54	55	56	57	58	59	60
VH4-39	W	G	W	I	R	Q	P	P	G	K	G	L	E	W	I	G	S	I	Y	Y	S	G	S	T	Y	Y	N
D1-10-N1	-	-	-	-	-	-	S	-	-	-	H	-	-	-	-	-	T	-	-	F	-	-	N	P	-	-	-
D2-14-N1	-	-	-	-	-	-	-	-	-	-	-	-	-	-	-	-	I	V	F	F	-	-	T	-	D	-	-
D3-12-N1	-	-	-	-	-	-	-	-	-	-	-	-	-	-	L	-	T	-	-	F	-	-	T	-	-	-	-
D3-23-N1	-	-	-	-	-	-	-	-	-	-	-	-	-	-	-	-	T	-	F	F	-	-	T	A	-	-	S
D3-24-N1	-	-	-	-	-	-	S	-	-	-	-	-	-	-	-	-	T	-	-	-	-	-	-	S	F	D	-
D3-31-N1	-	-	-	-	-	-	T	-	E	R	N	-	-	-	-	-	I	-	-	F	-	-	T	-	-	-	-
D4-10-N1	-	-	-	-	-	-	-	-	-	-	-	-	-	-	-	-	T	M	F	F	-	-	T	-	-	-	-

Kabat Numb.	61	62	63	64	65	66	67	68	69	70	71	72	73	74	75	76	77	78	79	80	81	82	82A	82B	82C	83	84	85	86	87	88
VH4-39	P	S	L	K	S	R	V	T	I	S	V	D	T	S	K	N	Q	F	S	L	K	L	S	S	V	T	A	A	D	T	A
D1-10-N1	-	-	-	Q	-	-	-	S	-	-	L	-	-	-	-	-	-	I	-	-	R	-	A	-	-	-	-	-	-	-	-
D2-14-N1	-	-	-	-	-	-	-	-	-	-	L	-	A	-	-	-	-	V	-	-	R	-	T	-	-	-	-	-	-	-	-
D3-12-N1	-	-	-	E	G	-	-	-	-	-	L	-	-	A	R	-	L	-	-	-	R	-	N	-	-	-	D	-	-	-	-
D3-23-N1	-	-	-	-	-	-	-	-	-	-	A	-	-	-	R	-	V	-	-	-	N	-	N	-	-	-	-	-	-	-	-
D3-24-N1	-	-	-	R	-	-	-	-	-	-	L	N	-	-	-	-	-	L	-	-	-	-	T	-	-	-	-	-	-	-	-
D3-31-N1	-	-	-	Q	-	-	-	S	M	-	L	-	R	-	T	-	R	-	-	-	R	-	-	-	-	-	-	-	-	-	-
D4-10-N1	-	-	-	H	-	-	-	-	-	-	A	-	-	-	-	-	H	-	-	-	R	V	T	-	-	-	-	-	-	-	-

													H3																						
Kabat Numb.	89	90	91	92	93	94	95	96	97	98	99	100	100a	100b	100c	100d	100e	100f	100g	100h	100i	100j	101	102	103	104	105	106	107	108	109	110	111	112	113
VH4-39	V	Y	Y	C	A	R																													
D1-10-N1	T	-	-	-	-	-	G	P	K	D	H	Q	P	.	.	.	.	.	.	.	.	L	Q	G	W	G	Q	G	T	L	V	T	V	S	S
D2-14-N1	-	-	-	-	V	-	P	T	Q	V	V	R	T	Q	P	G	P	.	.	.	.	F	D	L	W	G	Q	G	T	M	V	T	V	S	S
D3-12-N1	-	-	-	-	-	S	P	G	T	S	H	D	T	N	G	Y	Y	.	.	.	.	F	A	Y	W	G	Q	G	S	T	V	T	V	S	S
D3-23-N1	-	-	-	-	-	T	P	A	I	D	Y	D	S	G	G	S	Y	.	.	.	.	F	D	I	W	G	Q	G	T	L	V	T	V	S	S
D3-24-N1	-	-	-	-	-	S	G	S	K	S	D	S	G	W	.	.	.	.	.	.	.	F	D	P	W	G	Q	G	T	L	V	T	V	S	S
D3-31-N1	I	-	-	-	-	K	R	Q	S	M	G	D	P	S	L	T	G	D	G	L	.	F	D	L	W	G	R	G	T	L	V	T	V	S	S
D4-10-N1	-	-	H	-	-	K	P	K	S	R	D	R	G	G	P	G	D	D	Y	F	G	M	D	V	W	G	Q	G	I	M	V	T	V	S	S

Supplementary Figure 9. Heavy chain variable region sequence alignment of IGVH4-39 derived antibodies that bind NEAT1 and IGVH1-69 derived antibodies that bind NEAT2.

a) Members of the IGHV1-69 derived NEAT2-binding antibodies. The germline amino acid sequence of IGHV1-69*01 is shown for comparison. b) Members of the IGHV4-39 derived NEAT1-binding antibodies. The germline amino acid sequence of IGHV4-39*01 is shown for comparison. The CDRs are highlighted in gray.

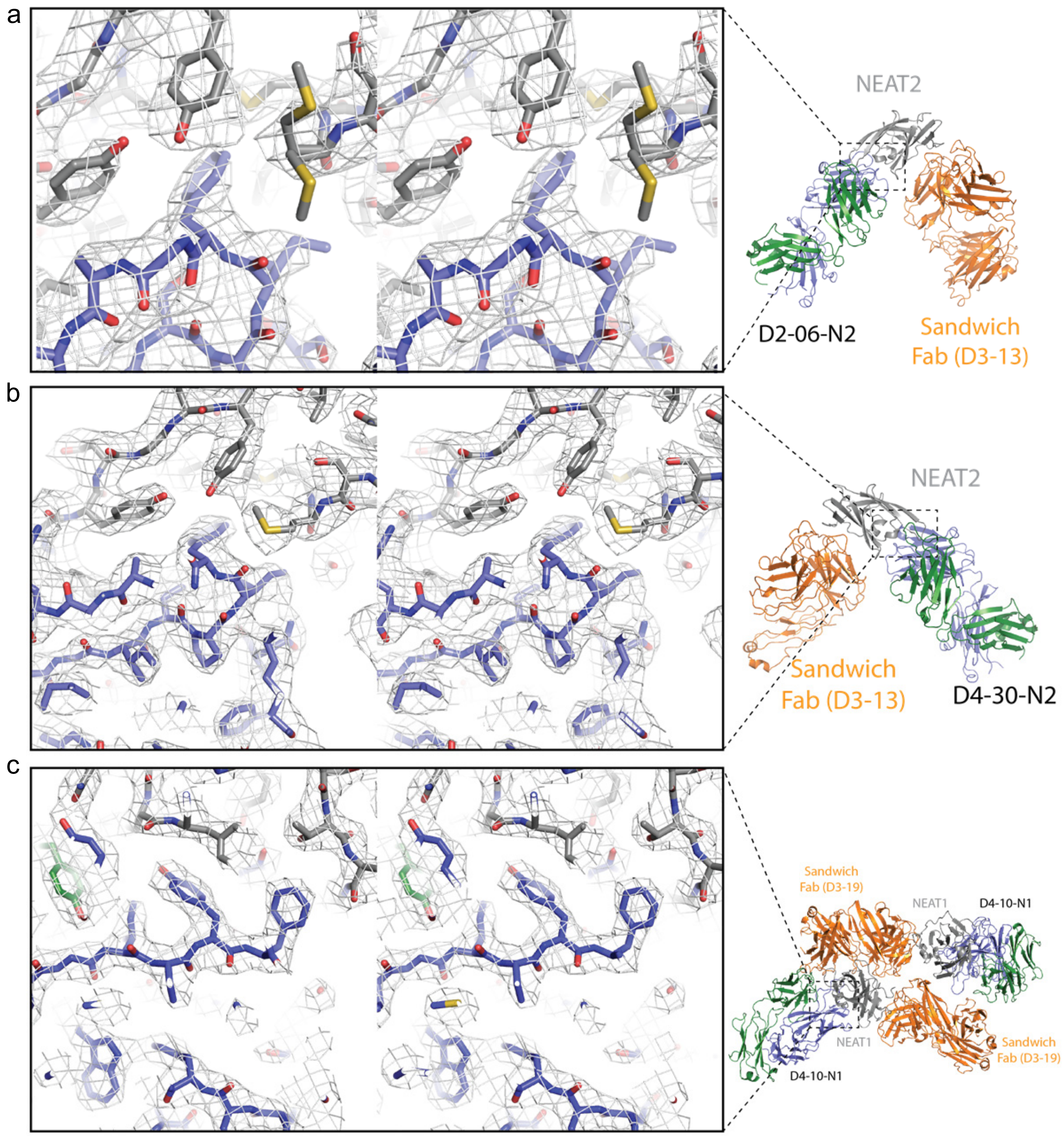

Supplementary Figure 10. Stereo images of the electron density maps and components within the asymmetric units.

a-b) D2-06-N2 and D4-30-N2 in complex with NEAT2 and the sandwiching Fab D3-13, represented in ribbon. Electron density map showing the fit for CDR-H2 (including the P52 I53 F54 motif) interacting with the residues that form the heme-pocket of NEAT2. c) Two D4-10-N1: NEAT1: sandwiching Fab D3-19 complexes are observed in the asymmetric unit, represented in ribbon. Electron density map showing the fit for CDR-H2 (including Y52 and F53) interacting with NEAT1 (including Y165 and H166).

a b

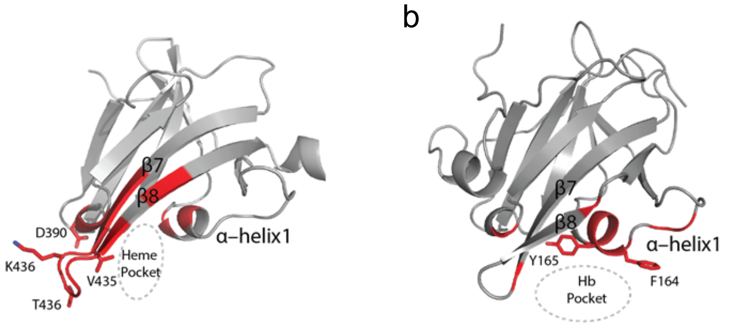

c

NEAT2	D1-N2-1	D2-N2-2	D2-N2-3	D3-N2-4	D3-N2-5	D3-N2-6	D3-N2-7	D3-N2-8	D4-N2-9	D4-N2-10	D4-53 (S)	D3-13 (T)	D4-62 (W)
	Structure							Structure			Positive Controls		
WT	100	100	100	100	100	100	100	100	100	100	100	100	100
M363R	100	100	100	100	100	100	100	100	100	100	100	100	100
F366R	52	42	36	52	7	26	3	25	40	11	88	91	87
D390A	33	113	96	88	4	3	111	80	111	121	104	106	103
D390R	2	48	28	2	3	2	23	15	105	63	103	106	109
Y391A	94	55	47	112	7	108	17	84	92	12	106	105	106
Y391R	85	81	3	41	3	39	3	17	84	23	104	105	107
K432A	91	91	87	95	87	87	80	89	86	79	97	98	97
V433R	62	44	27	55	3	8	2	15	39	11	93	93	93
H434A	97	100	100	99	112	100	102	105	98	102	103	103	100
V435R	2	3	3	5	3	2	3	3	5	3	96	96	97
K436A	105	110	105	102	78	102	116	112	3	47	104	103	105
T437A	90	84	95	97	38	93	18	6	83	8	96	100	98
T437R	2	78	3	20	3	3	2	2	2	2	105	104	108
I438A	93	88	49	93	68	99	99	6	73	5	102	102	101
I438R	48	3	3	5	3	2	3	3	3	2	97	99	100
D439A	102	113	111	106	123	104	113	113	105	121	104	105	103
Y440R	89	57	80	87	46	85	20	69	54	39	85	90	90
D441A	102	111	105	105	120	106	117	115	109	120	101	103	101
Y444R	84	69	70	82	55	74	49	63	65	43	93	92	92
Q443A	108	119	111	107	127	111	121	114	113	122	104	104	105
D390A/Y391A	3	4	7	3	5	3	6	20	64	9	100	104	103
D390A/K436A	5	68	3	4	3	3	64	4	83	4	98	99	97
D390A/T437A	3	5	5	5	3	2	3	2	55	2	94	97	97
D390A/I438A	2	5	3	3	3	2	29	3	13	3	80	87	85
Y391A/Y440A	75	44	37	78	3	66	9	55	57	6	80	85	82
Y391A/Y444A	72	3	3	73	3	46	2	9	7	2	81	94	94
H434A/K436A	79	51	59	84	60	66	60	64	65	7	92	91	90
H434A/D439A	70	65	68	71	63	67	38	63	61	37	95	96	94
H434A/D441A	74	77	75	78	68	74	57	71	71	51	85	93	92
K436A/437A	96	11	76	99	3	92	25	3	45	2	96	101	99
K436A/D439A	81	67	76	81	39	71	58	75	70	8	98	96	96
T437A/I439A	40	33	17	85	17	93	33	3	4	4	100	102	104
T437A/D439A	85	85	91	90	56	91	8	7	89	5	94	97	95
D439A/D441A	71	63	71	74	70	75	44	70	65	39	86	92	91
D390A/K436A/T437A	3	3	3	3	3	2	3	2	5	2	93	98	95
D390A/H434A/K436A	3	25	3	15	3	2	17	7	55	5	92	93	91
H434A/D439A/D441A	66	62	63	69	65	65	33	67	60	33	87	91	90
H434A/K436A/D439A/D441A	70	45	50	72	67	60	35	63	55	6	84	89	88
D390A/H434A/K436A/T437A	3	3	3	4	3	2	3	2	7	2	91	95	95

d

NEAT1	D1-N1-1	D2-N1-2	D3-N1-3	D3-N1-4	D3-N1-5	D3-N1-6	D4-N1-7	D2-02 (A)	D4-22 (D)	D4-09 (G)
							Structure	Positive Controls		
WT	100	100	100	100	100	100	100	100	100	100
F164A/Y165A/Y167A	3	3	2	3	3	2	3	98	98	98
F164A/Y167A	3	3	2	3	2	2	3	99	98	98
Y165A/Y167A	3	8	25	100	22	3	78	96	99	97
T161A	96	90	96	97	97	97	100	98	97	97
Q162A	98	99	97	98	100	99	95	99	98	97
F164A	103	96	95	93	101	106	96	97	96	97
F164R	5	96	112	98	105	4	97	99	96	96
Y165A	8	108	22	106	103	3	95	98	99	99
Y165R	4	3	3	3	7	3	4	98	97	98
H166A	113	101	101	94	101	111	99	ND	96	96
H166R	7	3	3	3	3	3	36	ND	99	100
Y167A	100	98	97	97	93	98	100	99	99	98
Y167R	99	94	96	93	96	95	97	98	97	94
A168G	103	98	100	98	98	105	98	99	99	99
A168R	6	98	98	106	6	8	96	99	96	96
F194A	106	97	100	90	92	99	94	ND	93	93
F194R	109	99	104	93	99	103	97	ND	96	97
F242A	88	101	70	100	107	105	98	ND	101	97
F242R	12	103	11	100	102	32	96	ND	99	99
Y249A	108	99	108	97	96	106	96	ND	96	93
Y249R	76	109	110	101	105	13	99	ND	100	100
F242A/N243A/N244A/K245A	104	97	98	103	100	102	97	98	98	99

%	Response
0-20	
21-50	
51-80	
>81	

Supplementary Figure 11. Mutagenesis at key IsdB positions supports the structural data and demonstrates analogous binding of all IGVH1-69 derived antibodies to NEAT2 and of all IGVH4-39 antibodies to NEAT1.

a-b) Mutations were introduced at selected positions of NEAT2 and NEAT1, and their effects on antibody binding were monitored by ELISA. Mutations of NEAT2 (a) are centered on the heme pocket while mutations of NEAT1 (b) are centered on the hemoglobin binding region. The mutations are colored in red on respective NEAT1 and NEAT2 structures. c-d) The binding responses to the NEAT1 and NEAT2 variants are normalized to the binding response to the wild type protein. Control antibodies which target non-overlapping epitopes on NEAT 1 and NEAT2 respectively, were also tested to ensure the mutation did not cause major disruptions or changes to the NEAT domain fold.

a

D2-06-N2
CDR-H3: CARDRPPGAQFDLW
CDR-L3: CQQANTFPWTF

Chain	Position	Amino Acid	BSA	% of Total BSA
H	95	D	11.0	1.4%
H	97	P	32.8	4.0%
H	98	P	35.1	4.3%
H	99	G	15.7	1.9%
H	100	A	31.7	3.9%
H	100b	F	4.1	0.5%
L	91	A	8.3	1.0%
L	92	N	12.1	1.5%
L	94	F	57.8	7.1%
L	96	W	54.4	6.7%

D4-30-N2
CDR-H3: CARDGRGALQYW
CDR-L3: CMQATHWPYTF

Chain	Position	Amino Acid	BSA	% of Total BSA
H	95	D	14.6	1.8%
H	96	G	1.4	0.2%
H	97	R	53.5	6.5%
H	98	G	8.5	1.0%
H	99	A	14.9	1.8%
H	100	L	6.8	0.8%
L	91	A	15.4	1.9%
L	92	T	7.5	0.9%
L	93	H	0.3	0.0%
L	94	W	76.8	9.4%
L	96	Y	57.4	7.0%

b

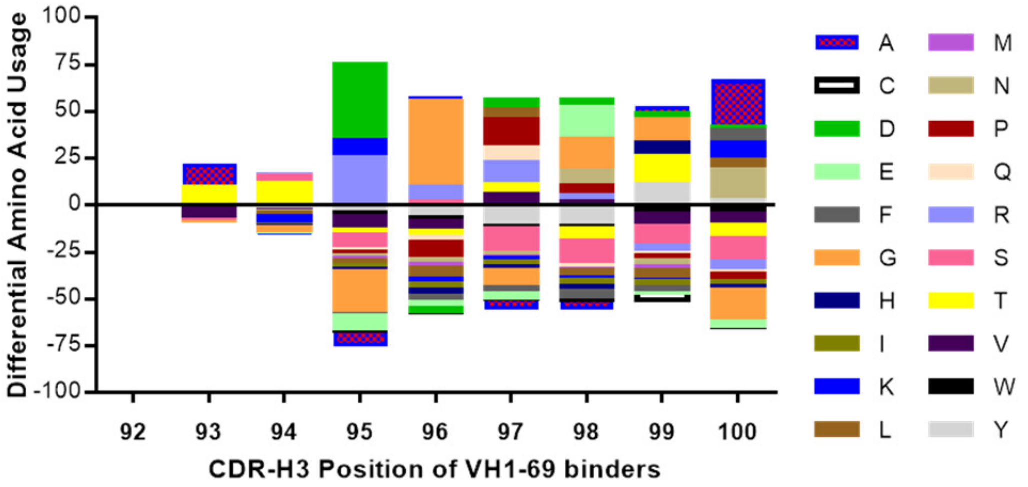

Supplementary Figure 12. Analysis of the binding contributions from residues in the CDR3 and J regions of IGVH1-69 derived NEAT2 binders

a) Analysis of the buried surface area (BSA) of the individual residues in the CDR3 and J regions based on the respective crystal structures of NEAT2 in complex with D2-06-N2 Fab and D4-30-N2 Fab. Only residues that contribute to BSA are shown. b) Comparison of positional amino acid variability of ten IGHV1-69 derived NEAT1 binders with the sequences of all IGVH1-69 derived antibodies in a control repertoire. The control repertoire was generated by sequencing the memory B cell repertoire of 12 healthy donors. Differential amino acid usages were tabulated by subtracting the usage percentage for each amino acid at each position in the control repertoire from that of the NEAT2 binders. Differential amino acid usage is shown for positions 92 to 100 of CDR-H3. Results show that cysteine at position 92 was almost equally represented in both NEAT2 binders and in the control repertoire. Results also show that charged residues (D, K and R) at position 95 and glycine at position 96 are overrepresented among the NEAT2 binders.

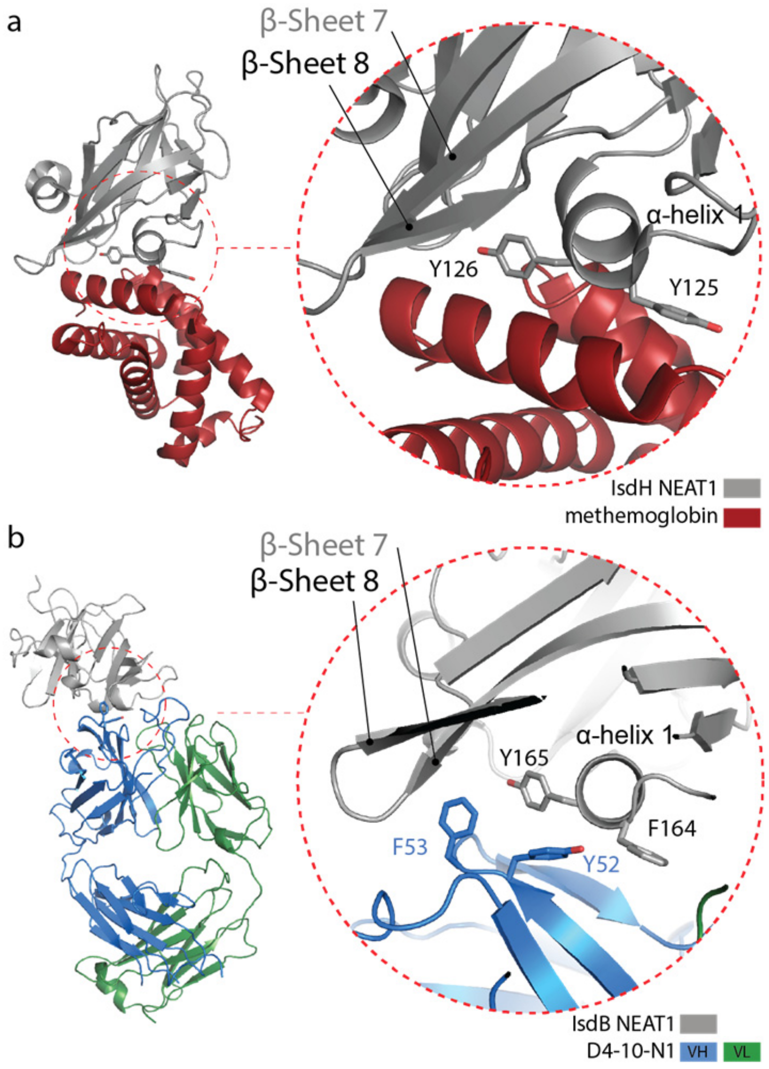

Supplementary Figure 13. Similar binding mode between IsdB NEAT1- D4-10-N1 and IsdH NEAT1-methemoglobin.

While there is no structure available for the IsdB NEAT1-hemoglobin complex, the interaction can be modeled using the solved structure of methemoglobin bound to IsdH NEAT1. a) The IsdH NEAT1-methemoglobin structure (PDB: 3SZK) shows that residues Y125 and Y126 of α–helix1 of IsdH NEAT1, which are structurally homologous to F164 and Y165 on IsdB NEAT1 α–helix1, are part of the hemoglobin-NEAT binding interface. A previous mutagenesis study also showed that IsdB NEAT 1 variants F164A, Y165A and Y167A completely lost hemoglobin binding[1]. b) The IsdB-antibody structure solved in this study shows that antibody D4-10-N1 targets the same α-helix region on the NEAT domain that is critical for hemoglobin binding.

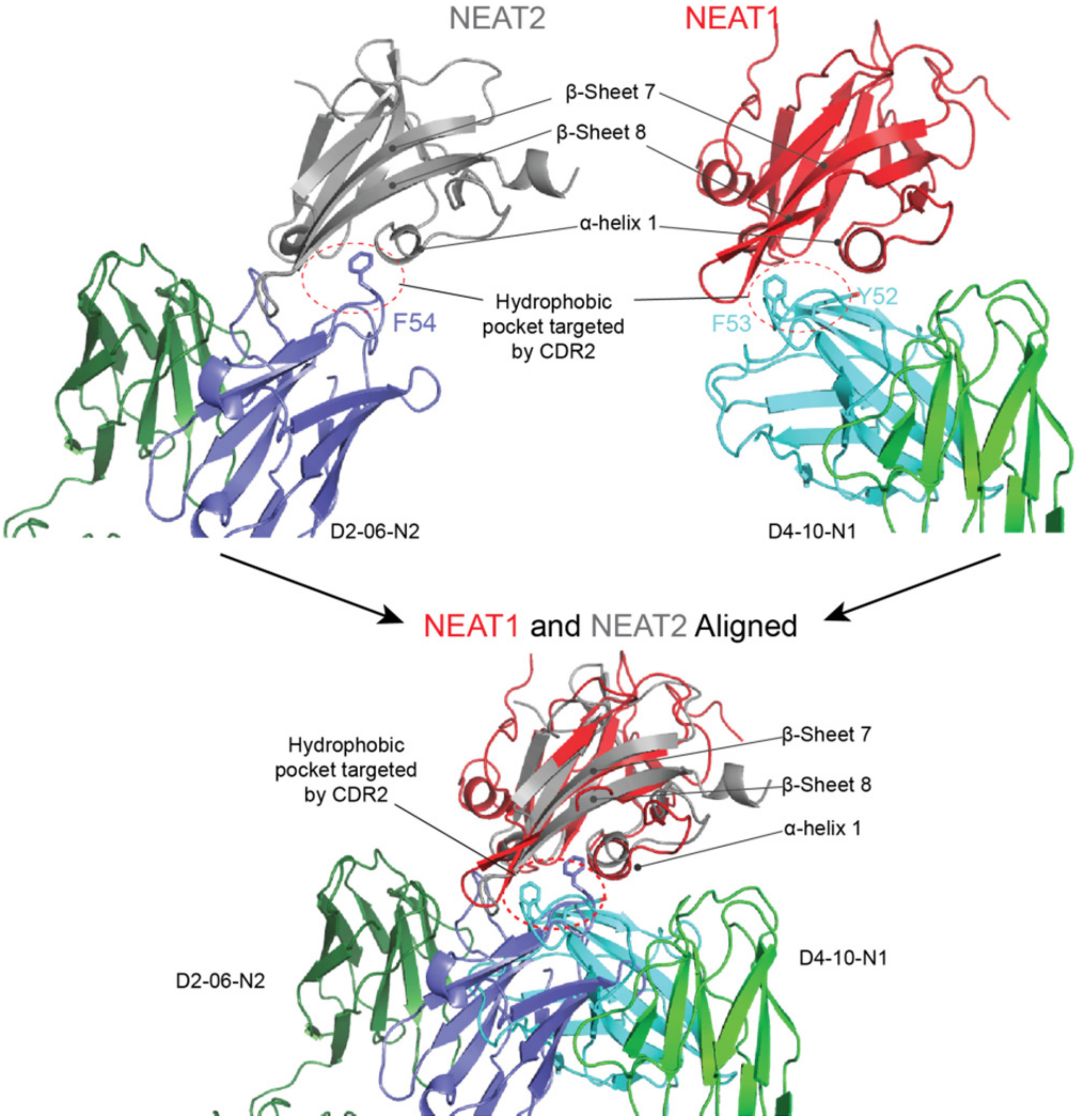

Supplementary Figure 14. CDR-H2 aromatic residues of D2-06-N2 (IGHV1-69) and D4-10-N1 (IGHV4-39) protrude into a structurally homologous region on the two respective NEAT domains.

The complex structures of D2-06/NEAT2 and D4-10/NEAT1 were aligned using the NEAT domain as reference. Despite targeting structurally similar NEAT domain folds, IGHV1-69 and IGHV4-39 derived antibodies are highly specific for their respective function-neutralizing epitope on the two NEAT domains. Fine differences in the sidechain identities and structural elements within the complexes likely explain the germline preference for antibody usage in binding the two NEAT domains of IsdB.

a

D4-10-N1
H3: CAKPKSRDRGGPGDDYFGMDVW
L3: CQQLNTYPFTF

Chain	Position	Amino Acid	BSA	% of Total BSA
H	98	R	21.1	2.6%
H	100a	G	0.3	0.0%
H	100b	G	2.6	0.3%
H	100c	P	37.2	4.6%
H	100d	G	4.7	0.6%
H	100e	D	46.8	5.8%
H	100g	Y	48.7	6.0%
L	91	L	8.5	1.0%
L	92	N	42.7	5.3%
L	93	T	9.7	1.2%
L	94	Y	31.1	3.8%
L	96	F	20.5	2.5%

b

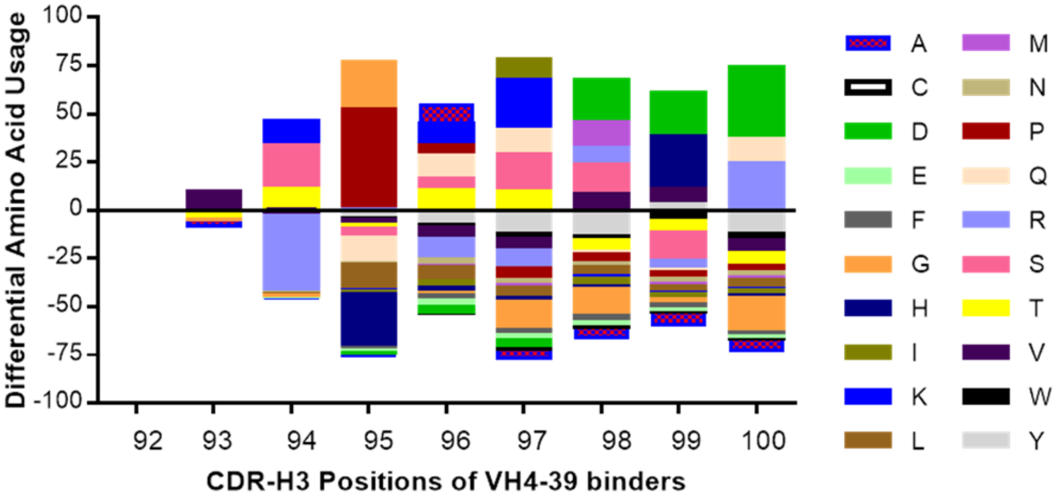

Supplementary Figure 15. Analysis of the binding contribution from residues in the CDR3 and J regions of IGVH4-39 derived NEAT1 binders

a) Analysis of the buried surface area (BSA) of the individual residues in CDR3 and J regions based on the crystal structure of D4-10-N1 Fab in complex with NEAT1. Only residues that contribute to BSA are shown. b) Comparison of positional amino acid variability of seven IGHV4-39 derived NEAT1 binders with the total IGHV4-39 derived antibodies in memory repertoire of 12 healthy donors. Results show that cysteine at position 92 was equally represented in both NEAT1 binders and control repertoire. Results also show that arginine at position 94 is underrepresented, while proline and glycine are overrepresented among the NEAT1 binders.

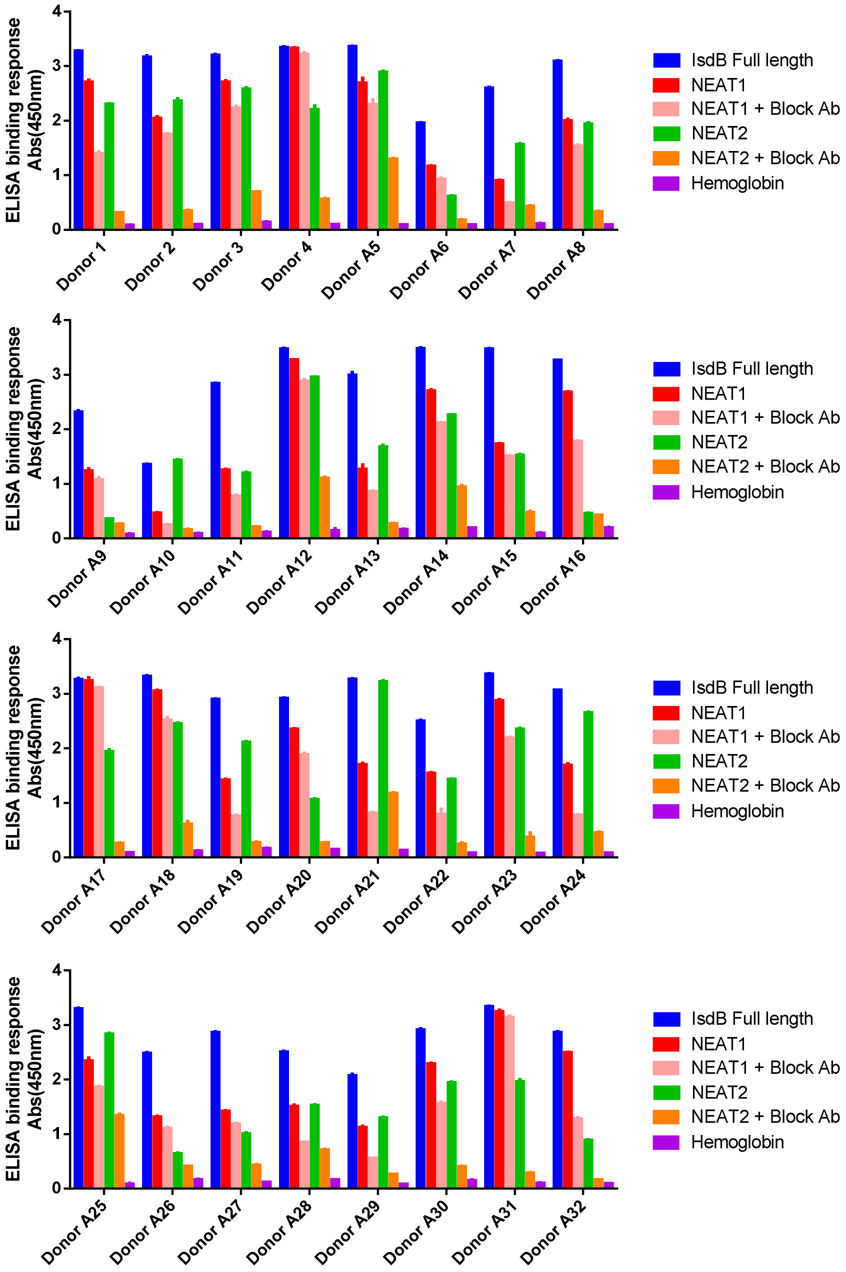

Supplementary Figure 16. Human serum titers against IsdB NEAT1 and NEAT2 domains.

Serum samples from 32 healthy donors (1-A32), plus those from Donors 1-4, were diluted 1:1000 and tested against recombinant full length IsdB, IsdB NEAT1, IsdB NEAT2 and human hemoglobin (negative control). Testing against IsdB NEAT1 and NEAT2 was also performed in the presence of functional epitope blocking antibodies D4-10 (NEAT1) and D2-06 (NEAT2) in mouse IgG forms, which were confirmed to not interfere with the anti-human IgG detection reagents. All the serum samples exhibited titers against NEAT1 and NEAT2, and their titers were reduced in the presence of blocking antibodies. Results shown are an average of three experiments. Error bars indicate standard deviations.

a

Donors Ab#	VH gene/ allele	VH FW mutation #	CDR-H3	CDR-H3 Length	DH gene / Allele	JH gene/ Allele	VK Germline	CDR-L3	JK Germline	Clone #
D6-1	IGHV4-39*01	1	CATYYYDSSGYYFPDAFDIW	20	IGHD3-22*01	IGHJ3*02	IGKV3-20	CQQYGSLYTF	JK2	2
D14-1	IGHV4-39*01	0	CASWGGNSGVDAFDIW	16	IGHD4-23*01	IGHJ3*02	IGKV3-20	CQQYGSSPLTF	JK4	646 (4 LC)
D15-1	IGHV4-39*01	0	CARPLTGTTHDAFDIW	16	IGHD1-20*01	IGHJ3*02	IGKV3-15	CQQYGGPLTF	JK4	77(2 LC)
D16-1	IGHV4-39*07	1	CARPHSSSYDSLGAFDIW	18	IGHD6-25*01	IGHJ3*02	IGKV3-20	CQQYGSSPLTF	JK4	102
D17-1	IGHV4-39*01	1	CASNRWELLRDRQAFDIW	18	IGHD1-26*01	IGHJ3*02	IGKV3-20	CQQYGSSPLTF	Jk5	3
D18-1	IGHV4-39*01	1	CAGIGSGSYYNPPDAFDIW	19	IGHD3-10*01	IGHJ3*02	IGKV3-15	CQQYGSSPLTF	JK4	7 (2 LC)
D18-2	IGHV4-39*01	0	CAGVDLIPPSGAFDIW	16	IGHD3-16*01	IGHJ3*02	IGKV3-20	CQQYGSSPITF	JK5	1
D18-3	IGHV4-39*01	0	CASRLPFIVGAPGAFDIW	18	IGHD1-26*01	IGHJ3*02	IGKV1-33	CQQSYSIPPSF	JK2	1
D20-1	IGHV4-39*07	1	CASGSPGGVAAFDIW	15	IGHD2-15*01	IGHJ3*02	IGKV3-20	CQQYGHSPLTF	JK2	3
D23-1	IGHV4-39*01	0	CAGGGQLHHHDAFDIW	16	IGHD2-15*01	IGHJ3*02	IGKV3-NL1	CQQYGSSPLTF	JK2	1
D27-1	IGHV4-39*07	0	CAGTSDRWLSGAFDIW	16	IGHD3-22*01	IGHJ3*02	IGKV3-20	CQQYGSSPLTF	JK4	2
D28-1	IGHV4-39*01	0	CAGRAGSGSFDDAFDIW	17	IGHD3-10*01	IGHJ3*02	IGKV3-20	CQQYGSSPYTF	Jk2	1
D29-1	IGHV4-39*01	0	CATRLCSGGSCYDDGAFDIW	20	IGHD2-15*01	IGHJ3*02	IGKV3-20	CQQYGSSPLTF	JK4	1
D32-1	IGHV4-39*01	0	CAGLSSSSGYANDAFDIW	18	IGHD3-22*01	IGHJ3*02	IGKV3-11	CQQYGSSPLTF	JK5	3
D40-1	IGHV4-39*01	0	CATYYGSGSGAFDIW	15	IGHD3-10*01	IGHJ3*02	IGKV3-20	CQQYGSSPLTF	JK4	9
D40-2	IGHV4-39*01	2	CATIPYTVGARIHAFDIW	18	IGHD1-26*01	IGHJ3*02	IGKV3-20	CQQYGSSPLTF	JK4	1

b

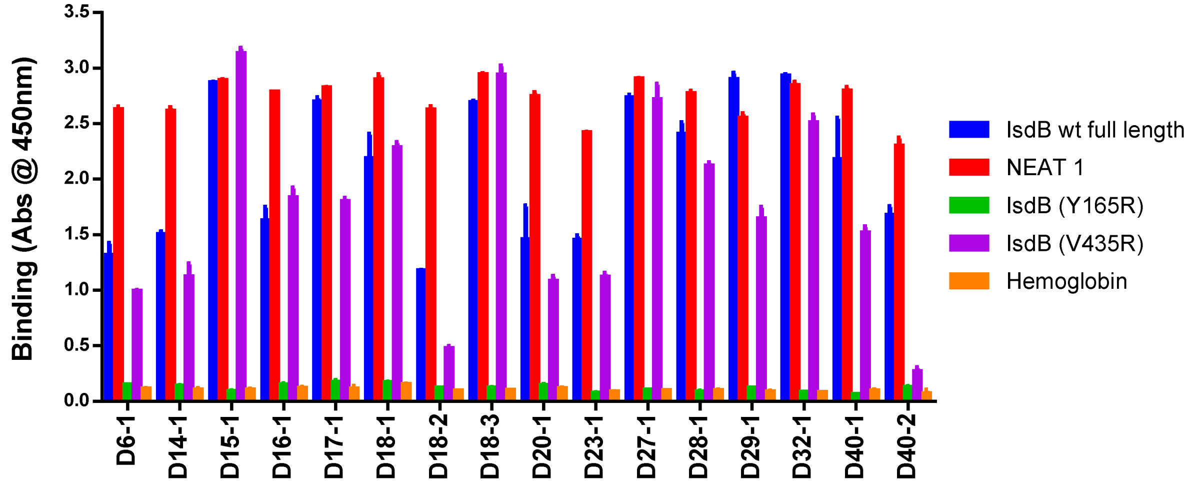

Supplementary Figure 17. All sixteen IGHV4-39 derived naïve antibodies isolated from the phage display library bind IsdB NEAT1, and all of them lose binding to the IsdB Y165R variant.

a) After 4 rounds of panning, sixteen NEAT1 binders with unique CDR-H3 sequences from 13 different donors were isolated. The V, D, & J germline usages, CDR3 identities and number of variable heavy chain framework nucleotide mutations of the 16 binders were tabulated using IMGT/V-Quest. The number of NEAT1-binding phage clones with identical CDR-H3 was shown. Three of the isolated unique phage clones based on CDR-H3 (D14-1, D15-1 and D18-1) were found to be paired with light chains of different germlines and CDR-L3. The most abundant light chain sequence for each unique phage clone was shown. b) Binding of the phage displayed antibodies in the format of single chain Fv against recombinant full length IsdB, IsdB NEAT1, IsdB Y165R variant and human hemoglobin, which served as a negative control antigen, were tested in ELISA. The signals shown are an average of three experiments. Error bars represent standard deviation of three experiments.

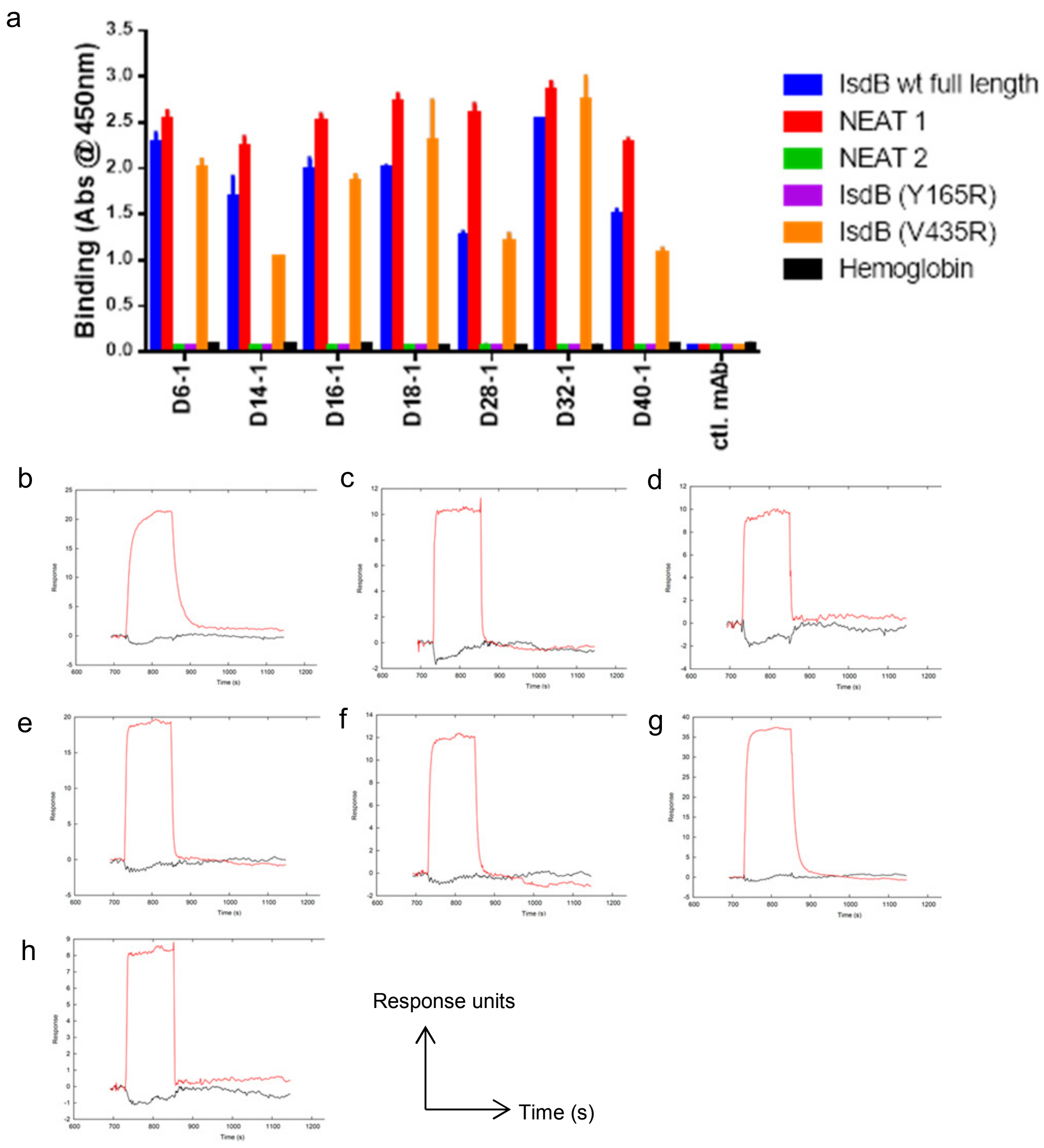

Supplementary Figure 18. Naive IGHV4-39 derived antibodies from naïve B cells bind NEAT1 in a similar manner as the antibodies isolated from memory B cells

a) Binding of seven reformatted IGHV4-39 derived antibodies, one each from a different donor, was tested by Elisa against recombinant full length IsdB, NEAT1, NEAT2, IsdB Y165R variant, IsdB V435R variant and human hemoglobin (negative control) . The signals shown are an average of two experiments. All the antibodies were able to bind full length IsdB, NEAT1 and IsdB V435R variant, while they lost binding to IsdB Y165R variant. b-h) Binding of the seven reformatted antibodies and their Y52A/Y53A variants against full length IsdB was also determined by SPR-based biosensor binding analysis at 37°C. Parental antibodies binding curves are shown in red, while binding curves of the Y52A/Y53A variant are shown in black. The antibodies shown here are b) D6-1, c) D14-1, d) D16-1, e) D18-1, f) D28-1, g) D32-1 and h) D40-1. Results shown are one experiment out of two independent runs.

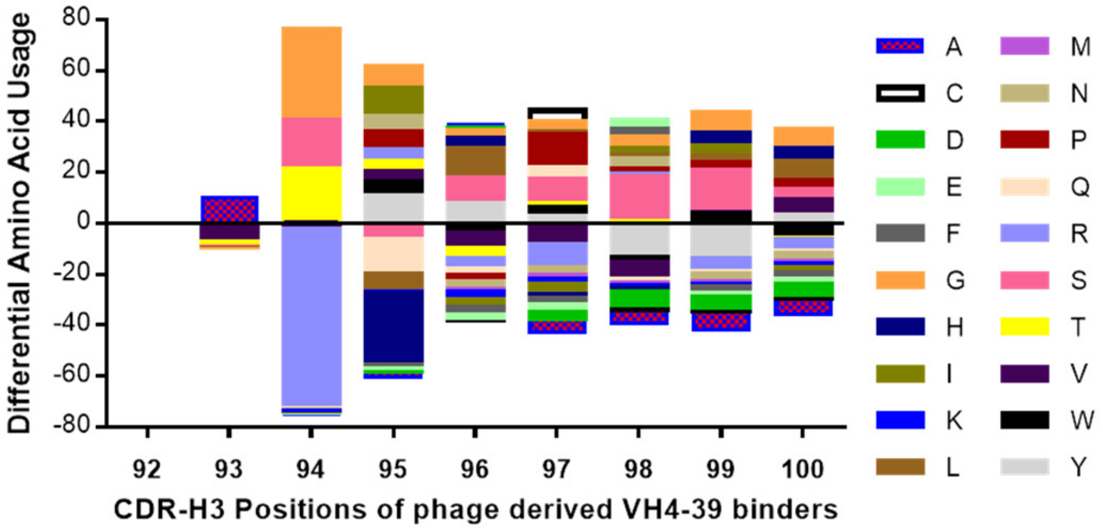

Supplementary Figure 19. Usages of CDR-H3 residues of IGVH4-39 derived NEAT1 binders from phage display

Comparison of positional amino acid variability of sixteen IGHV4-39 derived NEAT1 binders from phage display with the total IGHV4-39 derived antibodies in memory repertoire of 12 healthy donors. Results show that arginine at position 94 is underrepresented.

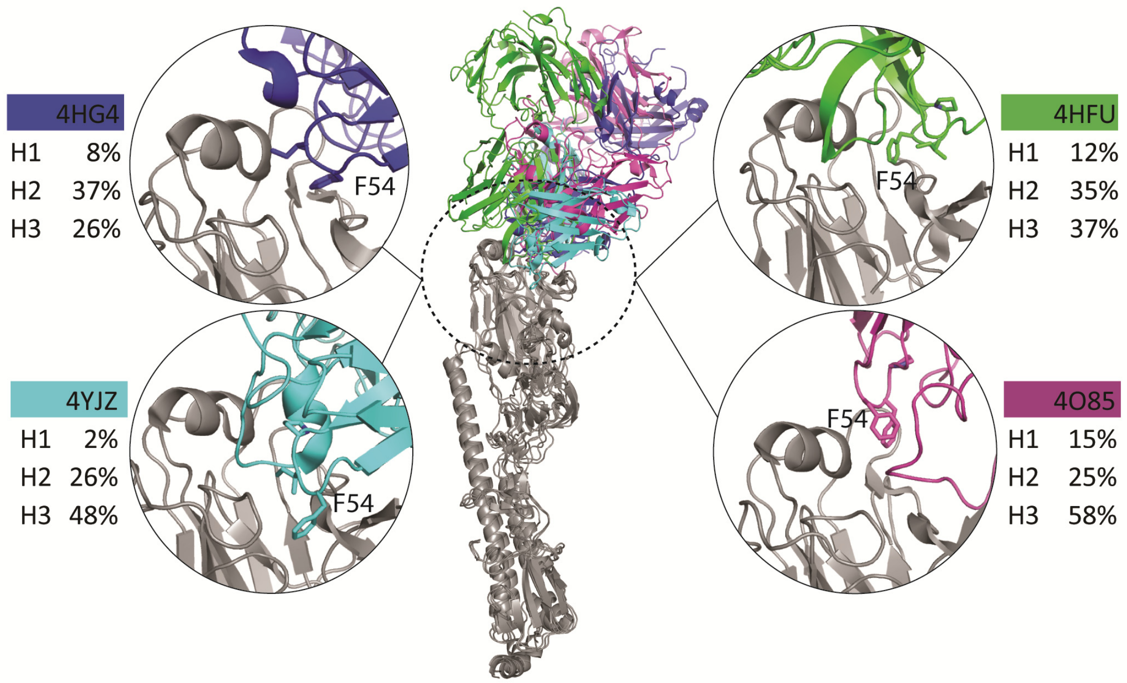

Supplementary Figure 20. The binding modes of IGVH1-69 derived antibodies against receptor binding sites on the head of HA are different

The structures of four IGVH1-69 derived antibodies bound to the receptor binding site of HA of the influenza virus were aligned based on HA (PDB: 4HFU, 4O85, 4HG4 & 4YJZ). The buried surface area contribution of the heavy chain CDRs is shown for each complex. All four VH1-69 antibodies target an overlapping region on the head of HA, suggesting a potential germline preference of VH1-69 antibodies for binding this region. However, comparison of the structural data shows that their binding modes are not identical. Three antibodies (4HFU, 4O85, 4HG4) do position F54 to target the hydrophobic cavity on HA, however most of the binding contributions are from CDR-H3, resulting in the antibodies having totally different binding orientations. The fourth VH1-69 antibody (4YJZ), instead of using F54, employs CDR-H3 hydrophobic residues to target the same HA cavity.

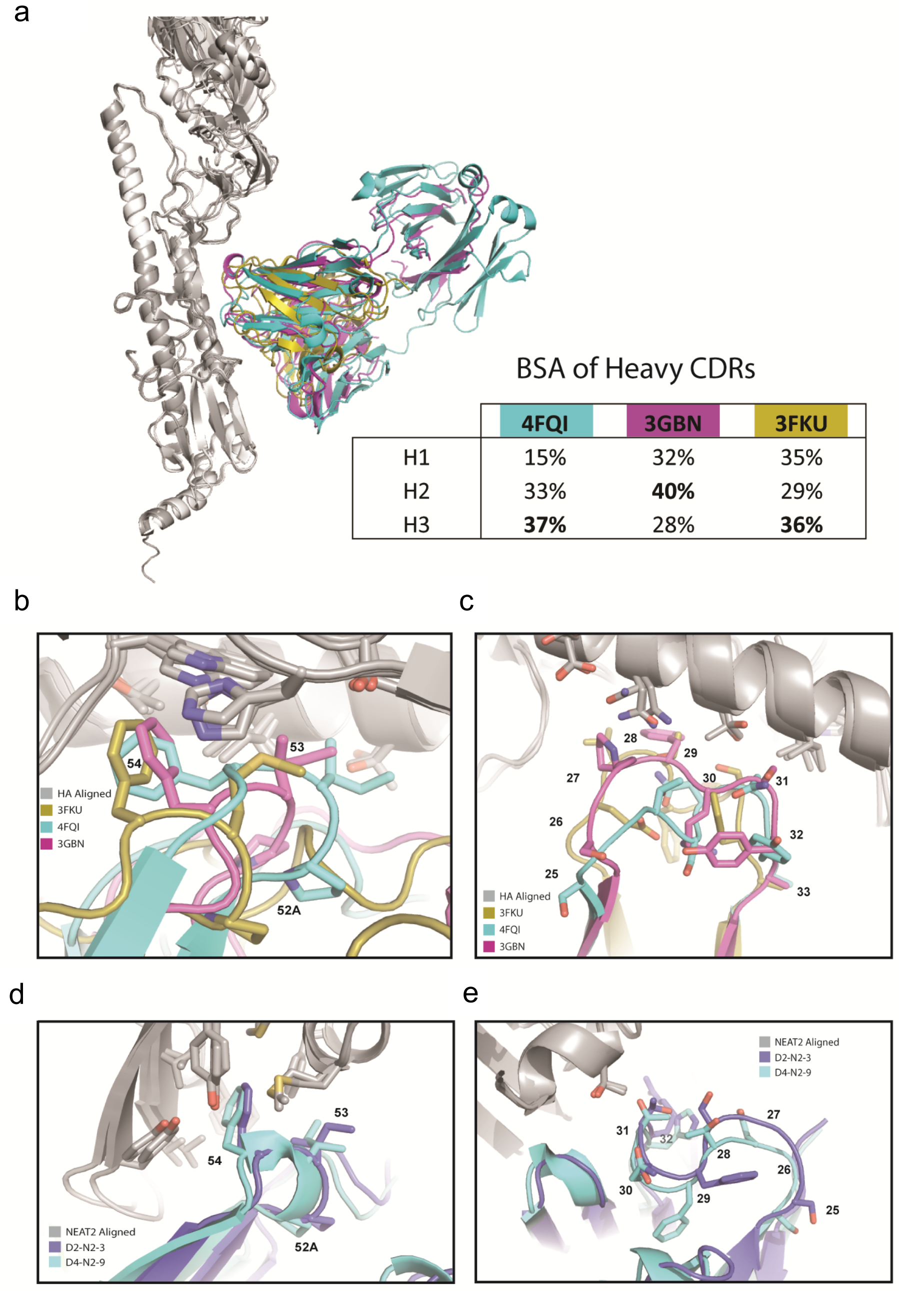

BSA of Heavy CDRs

	4FQI	3GBN	3FKU
H1	15%	32%	35%
H2	33%	**40%**	29%
H3	**37%**	28%	**36%**

Supplementary Figure 21. The binding modes of IGVH1-69 derived antibodies against the stem region of HA and IsdB NEAT2 domain.

a-c) Three complex structures (3FKU, 3GBN & 4FQI) of IGVH1-69 derived antibodies targeting a conserved blocking epitope on influenza virus HA were aligned based on HA. The buried surface area contributions of the three variable heavy chains are shown in (a). CDR-H1 and CDR-H2 loops for each complex are shown in (b) and (c), respectively. d-e) Two complex structures of VH1-69 derived antibodies targeting the heme-binding epitope on NEAT2 were aligned based on the NEAT2. CDR-H1 and CDR-H2 loops for each complex are shown. Residues are numbered based on Kabat

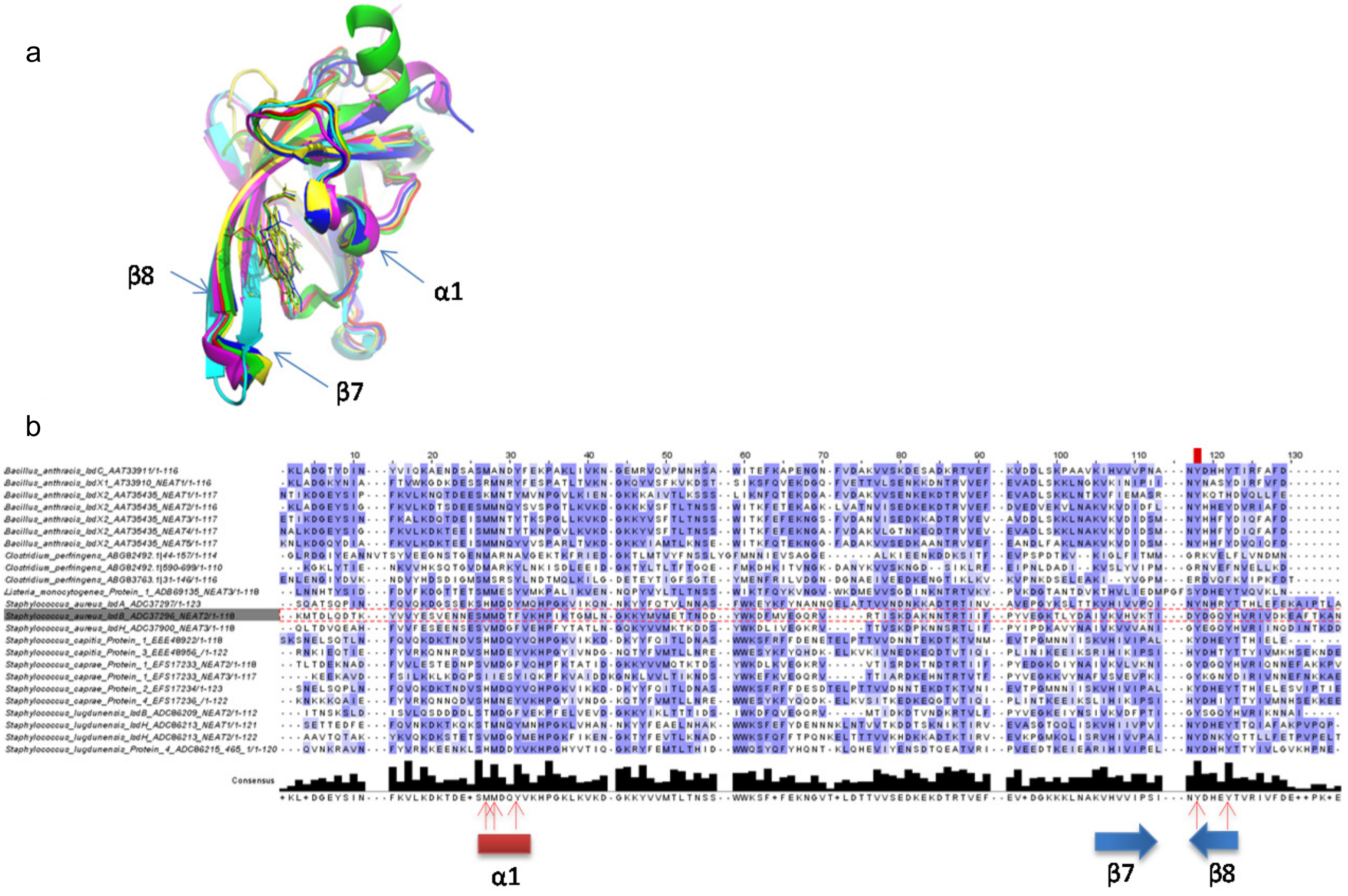
a
β8
α1
β7
b
Consensus
α1
β7
β8

Supplementary Figure 22. Alignment of structurally-verified or putative heme-binding NEAT domains from *Bacillus anthracis*, *Clostridium perfringens, Listeria monocytogenes*, *Staphylococcus aureus*, *Staphylococcus capitis*, *Staphylococcus caprae*, and *Staphylococcus lugdunensis.*

a) Structural alignment of heme-binding NEAT domains from *S. aureus* IsdA (yellow; PDB: 3QZO), *S. aureus* IsdB (green; PDB: 3RTL), *S. aureus* IsdH (red; PDB: 3VTM), *B. anthracis* IsdX1 (magenta; 3SIK), *B. anthracis* IsdX2 (blue; PDB: 4H8P), and *L. monocytogenes* Hbp2 (cyan; PDB: 4MYP). b) The structurally-verified or putative heme-binding NEAT domains from various bacteria (including those represented in panel a) were sequence aligned based on critical structural residues for the NEAT domain fold using MUSCLE. The residues are colored according to the percentage identity (BLOSUM 62 matrix) relative to consensus residue for each position within the set. Amino acid positions analogous to the hydrophobic heme pockets residues M362, M363, F366, Y440 and Y444 of *S. aureus* IsdB are highlighted with red arrows.

Supplementary Table 1. Primers for amplifying the variable region of immunoglobulin genes from single B cell

**Reverse transcription primers**			
IgG	GTGTGCACGCCGCTGGTC	IgK	TGGAGGGCGTTATCCACCTTCC
IgA	GTGGGAAGTTTCTGGCGGTCAC	IgL	GTGCTCCCTTCATGCGTGACC
**1st PCR leader region forward primer mix**			
1	CCATGGACTGGACCTGGAG	14	GCTTCCTCCTCCTTTGGATCTCTG
2	ATGGACATACTTTGTTCCACG	15	CTSCTGCTCTGGGYTCC
3	ATGGAGTTTGGGCTGAGCTGG	16	GTCCTGGGCCCAGTCTG
4	ATGGAATTGGGGCTGAGCTG	17	CCTGGGCTCTGCTSCTCCTC
5	ATGGAGTTGGGACTGAGCTG	18	GTTCTGTGGTTTCTTCTGAGCTG
6	ATGGAACTGGGGCTCCG	19	GTGGCCTCCTATGWGCTGAC
7	ATGAAACACCTGTGGTTCTTCC	20	ACAGGGTCTCTCTCCCAG
8	ATGGGGTCAACCGCCATC	21	ACAGGTCTCTGTGCTCTGC
9	ATGCAAGTGGGGGCCTC	22	ATTCYCAGRCTGTGGTGAC
10	ATGTCTGTCTCCTTCCTCATC	23	GCTCACTGCACAGGTTCTTGG
11	GCTCAGCTCCTGGGGCT	24	TCCCTCTCSCAGSCTGTG
12	CTTCCTCCTGCTACTCTGGCTC	25	CAGTGGTCCAGGCAGGG
13	TTTCTCTGTTGCTCTGGATCTCTG		
**1st PCR constant reverse primer**			
IgG	GCCTGAGTTCCACGACACC	IgK	CTGTACTTTGGCCTCTCTGGGATAG
IgA	CTG TCC GCT TTC GCT CCA G	IgL	TTCCACTGCTCRGGCGTCAG
**Nested PCR heavy chain variable region forward primers**			
1	CAGGTSCAGCTGGTGCAGTCTGG	5	CAGGTGCAGCTGCAGGAGTCGG
2	CAGGTCACCTTGAAGGAGTCTGGTCC	6	CAGGTGCAGCTACAGCAGTGGG
3	GAGGTGCAGCTGGTGGAGTCTGG	7	GARGTGCAGCTGGTGCAGTCTGG
4	GAGGTGCAGCTGTTGGAGTCTGG	8	CAGGTACAGCTGCAGCAGTCAGG
**Nested PCR heavy chain constant reverse primers**			
IgG	GGGAAGTAGTCCTTGACCAGGC	IgA	TCACACTGAGTGGCTCCTGG
**Nested PCR light chain variable region forward primers**			
1	CAGTCTGTGYTGACKCAGCC	9	CAGGCAGGGCTGACTCAGC
2	CAGTCTGCCCTGACTCAGCC	10	GACATCCAGWTGACCCAGTCTCC
3	TCCTATGAGCTGACWCAGCCA	11	GATATTGTGATGACCCAGWCTCCACTC
4	TCTTCTGAGCTGACTCAGGACC	12	GAAATTGTGTTGACRCAGTCTCCAG
5	CAGCYTGTGCTGACTCAATC	13	GACATCGTGATGACCCAGTCTCC
6	CWGSCTGTGCTGACTCAGCC	14	GAAACGACACTCACGCAGTCTCC
7	AATTTTATGCTGACTCAGCCCCAC	15	GAAATTGTGCTGACWCAGTCTCCAG
8	CAGRCTGTGGTGACYCAGGAG	16	GACATTGTGCTGACCCAGTCTC
**Nested PCR light chain constant reverse primers**			
IgK	GCACACAACAGAGGCAGTTCCAG	IgL	TGCTGGCCGCRTACTTGTTGTTG

References

1 Pishchany, G. *et al.* IsdB-dependent hemoglobin binding is required for acquisition of heme by Staphylococcus aureus. *J Infect Dis* **209**, 1764-1772, (2014).
